# Genome-wide association study of agronomic traits in winter wheat (*Triticum aestivum* L.) using a custom SNP marker set

**DOI:** 10.1186/s12870-025-07322-y

**Published:** 2025-10-01

**Authors:** Mikhail Bazhenov, Ludmila Bespalova, Mariia Samarina, Nadezhda Polevikova, Elena Agaeva, Artyom Debeliy, Alina Beliakova, Aleksandr Ponisko, Lyubov Nazarova, Anastasiya Chernook, Gennady Karlov, Mikhail Divashuk

**Affiliations:** 1 All-Russia Research institute of Agricultural Biotechnology, 42 Timiryazevskaya Street, Moscow, 127550 Russia; 2 Department of Breeding and Seed Production of Wheat and Triticale, National Center of Grain Named after P.P. Lukyanenko, Central Estate of KNIISH, Krasnodar, Russia

**Keywords:** Winter wheat, Targeted genotyping by sequencing, SNP panel, GWAS, Agronomic traits, Population structure, 1RS.1BL translocation

## Abstract

Targeted genotyping by sequencing (GBS) of single-nucleotide polymorphisms (SNPs) is a relatively new high-throughput genotyping technique that combines target enrichment using polymerase reaction, followed by high-throughput sequencing of the resulting DNA fragments. Unlike traditional GBS, this approach requires prior knowledge of polymorphisms in the genome. Based on annotated polymorphisms in a wheat reference genome, we developed a panel of SNPs suitable for targeted GBS. Initially, 60,000 SNPs were proposed, including those tested by breeding markers, SNPs mentioned in previous genome-wide association studies (GWAS) publications, as well as mutations in protein-coding regions of genes. Due to the polyploidy of the wheat genome, the suboptimal GC content in surrounding regions and the requirements for high-quality allele calls, only 3500 SNP were retained for further analysis. These SNPs were used to genotype 200 winter wheat accessions maintained at the National Grain Center in Krasnodar, Russia. A population structure analysis based on genome-wide SNP data revealed two subpopulations in the collection corresponding to domestic and foreign cultivars. Genotyping results for specific SNPs revealed genes whose diversity can be enriched in the domestic gene pool to improve grain size (*TaGW2-A1*) and protein content (*Gpc-B1*). Additionally, no-call variants of SNPs obtained from SNP-seq procedures were used to detect chromosome rearrangements such as 1RS/1BL wheat-rye translocations. GWAS using mixed linear models identified new marker-trait associations for days to heading, plant height, visual grain score and disease resistance. KASP markers were developed for significantly associated SNPs and candidate genes based on their proximity and function were suggested. Additionally, a potential link was proposed between the presence of awns and increased calcium in grain. The GWAS also confirmed the known effects of reduced height (*Rht*) and photoperiod sensitivity (*Ppd*) genes in wheat, and the *Ppd-D1* gene showed an almost significant association with grain yield. SNPs originating from breeding markers were the most effective in finding marker-trait associations, while those from other sources found associations as frequently as random SNPs. Of the 13 markers significantly associated with traits, seven did not overlap with markers from existing genotyping arrays, and could be considered novel.

**Supplementary Information:**

The online version contains supplementary material available at 10.1186/s12870-025-07322-y.

## Introduction

The DNA molecular markers have been used for a long time in plant breeding to assess genetic diversity in collections, determine kinship between accessions, select parent lines for hybridization, evaluate breeding material, and implement marker-assisted selection [1]. The modern high-performance genotyping platforms allow simultaneous coverage of several thousand markers. High-performance data can be used to accurately map quantitative trait loci (QTL) in hybrid populations or for genome-wide association studies (GWAS), with the prospect of discovering candidate genes and functional polymorphisms that cause variations in valuable traits [2]. Also, high-performance genotyping data can be used for genomic selection. This is a method of selection that uses predicted values of breeding lines based on marker information and statistical or machine learning models. Genomic selection improves the efficiency of breeding for yield, disease resistance, product quality, and other traits [3, 4].

For wheat, several genotyping arrays have been developed. These include the 90 K iSelect Array from Illumina, which has 81,587 single nucleotide polymorphisms (SNPs) [5]; the 35 K Axiom Wheat Breeder’s Array, which contains 35,143 SNPs [6], and the 820 K Axiom wheat genotyping array, with 817,000 SNPs [7]. The most modern one is the Axiom ‘Triticum aestivum Next Generation’ array (TaNGv1.1), which can detect 43,373 selected polymorphisms [2]. Alternatives to DNA arrays include high-performance sequencing technologies such as genotyping by sequencing and targeted genotyping by sequencing (SNP-seq).

Genotyping by sequencing involves the cleavage of genomic DNA using restriction endonucleases, followed by the ligation of adapters and DNA barcodes. PCR amplification is then used to amplify the DNA fragments, which are subsequently sequenced using high-throughput methods [8]. Genotyping by sequencing can be used for those species whose genome has not been sequenced before [9].

Targeted genotyping by sequencing of single nucleotide polymorphisms (SNP-seq) is a newer technology that combines multiplex polymerase chain reaction (PCR) with two primers flanking each SNP or, alternatively, single primer enrichment technology (SPET), followed by high-throughput sequencing of the resulting amplified products [10, 11]. This approach is suitable only for species whose genomes have been previously sequenced and information on polymorphisms is available. Information on SNPs can be obtained from transcriptomes, exomes, or whole genome resequencing of several or many individuals. These limitations explain why targeted genotyping by sequencing has only recently been used in genome-wide studies. However, this approach offers great opportunities for researchers to choose polymorphisms for genotyping and may be more cost-effective than DNA arrays. The SNP-seq method has been successfully used for high-throughput genotyping of a variety of crops, including eggplant [12], cucumber [10], watermelon [13], melon [14] and pepper [15, 16]. In wheat this method was first applied in 2018, when a set of capture probes for targeted genotyping by sequencing was designed based on publicly available probes from Axiom arrays and KASP (competitive allele specific PCR) markers [17].

The Lukyanenko National Grain Center (LNGC) in Krasnodar, Russia, is a leading state-owned breeding institution that has been specializing in the development of winter wheat varieties for more than 100 years [18]. Winter wheat varieties developed by LNGC account for approximately 30% of total area under winter wheat cultivation in Russia and approximately 50% variety names on the list [19]. These varieties are extensively grown in southern regions of Russia as well as in Transcaucasia and Central Asia abroad. The widely known cultivar Bezostaya 1, developed here by Academician Pavel Lukyanenko in the 1950s, has become one of the most significant sources for genetic material from which many modern wheat varieties have been derived [20].

The aim of this study was to create a panel of polymorphisms that could be used for targeted genotyping by sequencing (SNP-seq) in common wheat. The idea behind using SNP-seq technology was to reduce genotyping costs while maintaining a high rate of discovery of marker-trait associations in GWAS. We assumed that if we used specific markers that were already associated with traits in other GWAS or SNPs in genes affecting protein function, we would be able to find more marker-traits associations at a lower cost. Our main focus was on finding associations that were directly applicable to the gene pool that we worked with, as SNP-seq markers could be easily converted into KASP markers and used for marker-assisted selection. Using a developed SNP set, we conducted a genome-wide association study (GWAS) for morphological and agronomic traits in a winter wheat collection maintained at LNGC, and explored the diversity of breeding material to identify chromosome loci essential for wheat adaptation to climate and soil conditions in southern Russia.

## Materials and methods

### Plant material

The collection of 200 winter bread wheat accessions (*Triticum aestivum* L.), including Russian and foreign cultivars and advanced breeding lines, was maintained and phenotyped during the growing seasons of 2018–2020 at the P.P. Lukyanenko National Grain Center in Krasnodar, Russia. Field experiment design, weather conditions and some characteristics of wheat accessions used in this study were published in our previous work [21, 22]. For the genome-wide association studies the phenotypes for the following traits were used: time from sowing to heading (days), plant height from the soil surface to the top of the spike excluding awns (cm), grain yield (t/ha), 1,000 kernel weight (g), test weight of grain (g/l); grain protein content, gluten content, and starch content (estimated using near-infrared spectroscopy on whole grains on an Infratec 1241 Grain Analyzer, Foss, Denmark); leaf rust, stripe rust, and Septoria blotch leaf damage, lodging score before harvest, grain score, and the presence of awns as a stable characteristic of the variety. Generally, the observations were done using the Methodology of State Variety Testing of Agricultural Crops [23]. An estimation of the percentage of leaf rust damage, as an average of damage to the two upper leaves, was made 10–12 days after heading, visually using a scale similar to the ‘USDA’ scale discussed by Peterson et al. [24]. The percentage of leaf area occupied by *Septoria* disease was also estimated visually on the two upper leaves without an external scale at milky ripeness. A visual estimation of lodging before harvest was done using a traditional scale of 1–9, similar to that described by Berry et al. [25]. On that scale, 9 means “no lodging”, 7 – plants are slightly lodged or stand upright after lodging, 5 – medium degree of lodging, 3 – plants lodged significantly making machine harvesting difficult, 1 – severe lodging making harvesting impossible. Other values denote intermediate states of lodging. The visual estimation of grain is done on a 1–9 scale, with 1 being an unsatisfactory grain appearance and 9 being an excellent grain appearance.

### SNP marker set development

The wheat reference genome sequence, IWGSC RefSeq v1.0 (Chinese Spring), and its annotation were used for the development of an SNP marker set [26, 27] (http://wheat-urgi.versailles.inra.fr/Seq-Repository/Annotations). In particular, polymorphisms revealed during sequencing of the exomes of 890 commercial and local wheat varieties [28], and varietal SNPs, were used as basis for selection. Among these polymorphisms, selection was done according to the following criteria, in descending order of preference: (1) polymorphisms that matched those detectable with KASP markers used in wheat breeding were selected first [29], (2) SNPs matching with polymorphisms detectable using the 90 K iSelect wheat DNA chip [5], which were mentioned in research articles (Additional file 1, Supplementary Table 1) were selected second, (3) SNPs located within wheat genes, the sequences of which were placed in the NCBI GenBank database, meaning that these genes were at some point in research focus, were selected third (4) SNPs annotated as functional polymorphisms causing missense, nonsense, or exon-intron boundary changing mutations in protein coding genes were selected fourth, (5) any other highly-variable polymorphisms were finally selected, to cover large gaps between the others and fulfil the total number of 60 K SNPs (Additional file 2).

Mapping KASP-detectable polymorphisms to the RefSeq1.0 wheat genome was done using in-silico PCR [30]. The names of 4,537 polymorphisms detectable with 90 K iSelect DNA array that previously showed a connection to agronomical traits were extracted automatically from the texts of the 20 articles and their supplementary materials (Additional file 1, Supplementary Table 1) using a Python script. Mapping sequences of selected probes from the 90 K iSelect array to the RefSeq1.0 wheat genome was done using Bowtie2 v2.5.4 software [31]. For each mapped position, the nearest SNP from the genome annotation was selected. Additionally, we extracted 5,417 records from the GenBank NCBI database (https://www.ncbi.nlm.nih.gov/nucleotide), containing wheat DNA sequences with lengths similar to genes (between 2 and 20 kilobases). Mapping these sequences to the wheat genome using Bowtie 2 [31] yielded 1,312 annotated gene identities for these records. Most GenBank records were mapped to some gene IDs, however 341 records had no gene IDs found. From these mapped ‘GenBank’ genes, we selected 3,500 polymorphisms to include in the marker set, with an average of 2–3 polymorphic sites per gene.

To find out how unique are the sequences surrounding each SNP we extracted the 101 bp SNP-containing fragments from RefSeq1.0 genome assembly (SNP nucleotide with ± 50 bp adjacent range), packed them into a FASTQ file with uniform highest quality scores using a custom python code and mapped them to the genome using BWA FASTMAP algorithm (BWA version 0.7.17-r1188) [32]. From the alignment file the longest exact match was selected for each SNP-containing 101 bp fragment and the number of hits was collected using a custom python code. Target enrichment oligonucleotide SPET probes, that were developed for seq-SNP experiment (designed by LGC Genomics GmbH, Germany), were located within SNP ± 100 bp fragments. One or two probes were designed for each SNP. The distances from probe ends to SNPs, GC nucleotide pairs content for probes and adjacent to SNP DNA fragments were calculated using custom python code. To find the positions of SNPs in a newer version RefSeq 2.1 genome, larger 201 bp fragments with ± 100 bp adjacent range were used for mapping the same way. The positions of SNPs in a newer genome version were calculated from alignment. Mapping positions, numbers of mapping hits and GC content for the SNP-adjacent fragments are available in Additional file 2, while for the oligonucleotide probes – in Additional file 3.

### Genotyping

For DNA extraction, 20 seedlings from each wheat accession were grown on wet vermiculite until they reached the stage of 2–3 green leaves. The seedlings were then cut and the plant material was mixed within each accession. It was dried and transferred to LGC Genomics GmbH, Germany (services in Russia distributed by MaxMedical LLC in 2019), for DNA isolation and SNP-seq genotyping.

The services provided by LGC Genomics GmbH included: verification of SNPs and SPET oligo probes design, synthesis of an oligo probe library, DNA extraction, quality control of extracted DNA, library preparation, including indexing and quality control, Illumina single-read sequencing with read length of 75 bp, raw read processing and quality trimming, sequence alignment using Bowtie 2 [31] and variant calling using freeBayes v1.2.0 [33]. The results of genotyping were provided in a variant-call format. The SNP-seq genotypes provided for each wheat accession represent an averaged genotype for several individual plants. Raw sequencing data, including non-trimmed reads, were deposited to NCBI SRA under accession number PRJNA1270396. The correspondence between sequencing samples and wheat accessions is provided in Additional file 8.

Additionally, DNA from two plants of each accession was extracted using a cetrimonium-bromide-based protocol [34]. This DNA was used for genotyping with polymerase chain reaction (PCR) markers of plant height genes *RHT-B1* (*Reduced height)* and *RHT-D1* alleles *Rht-B1a* (tall), *Rht-D1a* (tall), *Rht-B1b* (semidwarf), *Rht-B1e* (semidwarf), and *Rht-D1b* (semidwarf) [35, 36], and photoperiod sensitivity gene *PPD-D1* (*Photoperiod)* alleles *Ppd-D1a* (photoperiod-insensitive) and *Ppd-D1b* (photoperiod-sensitive) [37]. The presence of 1RS.1BL translocation was confirmed using PCR with simple-sequence repeat marker SCM9 located on 1RS rye chromosome arm [38]. The PCRs were conducted under conditions provided in original articles describing the markers. The PCR products were detected under ultraviolet light after electrophoresis in 1.5–2% agarose gel with Tris – boric acid – EDTA buffer with addition of ethidium bromide.

For those SNP-seq markers which were significantly associated with traits, the primers for KASP were designed based on reference genome sequences Chinese Spring (IWGSC RefSeq v1.0) using online tools for calculating melting temperature (https://bsu.bio/molbiol/oligocalc.html) and assessing potential non-specific hybridization of primers (https://www.unafold.org/Dinamelt/applications/two-state-melting-hybridization.php). The PCR mixture included 5 µl of the master mix, containing FAM, HEX and ROX fluorescent dyes (KASP TF V4.0 2X Master Mix, KBS-1050-102, LCG Biosearch Techhnology, Teddington, UK), 5 µl of matrix DNA at a concentration of 15–30 ng/µl, and 0.14 µl primer mix for each SNP (Additional file 1, Supplementary Table 2).

The PCR conditions for KASP markers were as follows: initial denaturation at 94 °C for 15 min; 9 cycles: denaturation at 94 °C for 20 s, annealing and elongation – 60 °C for 1 min, with a decrease in temperature by 0.6 °C each cycle; 35 cycles: denaturation at 94 °C for 20 s, annealing and elongation at 55 °C for 1 min; cooling to 37 °C for 1 min to read the fluorescence signal. The CFX96 amplifier device (BIO-RAD, Hercules, USA) and CFX Manager software were used to perform reaction. Examples of allele-discriminating diagrams are shown in Additional file 1, Supplementary Fig. 7.

### Data Preparation

The data were processed using TASSEL 5.2.88 software [39]. Initial genotype data were filtered using the following conditions: each marker should be successfully genotyped in 90% or more accessions, the minor allele frequency should be greater than 0.05, and the heterozygous state of each marker should not be observed in more than 10% of accessions. Missing genotypes were imputed using the LD-kNNi method (Linkage Disequilibrium – k-Nearest Neighbor Imputation) with default parameters (high LD sites is 30, the number of nearest neighbors is 10 and the max distance between sites to find LD is 10^6^) [40]. The best linear unbased estimations (BLUEs) for the quantitative agronomical traits based on three-years phenotypes were calculated in TASSEL 5.2.88 (Additional file 5).

### Population structure analysis

Structure 2.3.4 software, implementing cluster analysis based on the Markov Chain Monte Carlo simulations, was used for population structure inference with admixture model [41]. The estimated number of subpopulations (K) ranged from 1 to 10, with 50,000 burn-in iterations and 50,000 recorded Markov chain iterations in 10 replicate runs. The results were collected for visualization using Structure harvester (2022) software [42]. The most probable number of subpopulations was estimated using the ΔK statistics [43]. The neighbor-joining clustering based on an identity-by-state distance matrix for 200 wheat accessions calculated using 3,456 polymorphic markers was done in TASSEL 5.2.88.

### Finding marker-trait-associations

For the discovery of marker-trait associations, mixed linear models (MLM) in TASSEL 5.2.88 were applied. To compensate for population structure, 10 first principal components (covering 34% of the total variance) and a kinship matrix calculated from genotype information were used. A kinship matrix was calculated using a centered IBS method [44]. An effective number of SNPs used for determining a new alpha level after a Bonferroni correction was determined using the SimpleM R script [45].

### Visualization

Boxplots, violin plots, Manhattan and QQ plots, principal components (PC) scatterplot were created using Python v3.11. Boxplots and scatterplot were created using the Matplotlib library; violin plots – using Seaborn library [46]; Manhattan and QQ plots were generated using the qmplot library v0.3.1 [47] with minor modifications (the highlighting of top points was done off). A color palette for plots was obtained from the Seaborn library [46]. The code used for data preparation is available in public repository (10.5281/zenodo.15490241) [48]. The marker map was generated using MG2C v.2.1 online tool [49] and then further modified using Inkscape 1.3.2 vector graphics editing software. The hierarchical neighbor-joining tree was drawn using FigTree v1.4.4 [50]. Population structure plot, ΔK plot and other visualizations were created in MS Excel 2019.

## Results

### SNP set development

The initial set of single-nucleotide polymorphisms (SNPs) selected from the wheat genome annotation included 65 that are most often applied for marker-assisted selection (MAS) being detected by KASP markers; 4,537 SNPs that were previously associated with traits in various GWAS studies using the iSelect 90 K array; 3,500 polymorphisms found within genes deposited in GenBank database; 26,149 SNPs in protein-coding genes predicted as nonsense (almost all available nonsense mutations were included) or missense mutations affecting protein functionality; and 25,750 highly-variable SNPs taken at about equal physical distances to cover large gaps between other markers. The total number of SNPs in initial set was approximately 60 thousand. For this article, SNP markers are designated with numbers ranging from 1 to 60,001, preceded by the abbreviation IAB, which stands for ‘Institute of Agricultural Biotechnology’ (Table 1, Additional file 2).

**Table 1:** Number of SNPs remaining after sequential stages of filtering

Basis of choice	Initial number (0)	Successful primer design (1)	Successful SNP calling in > 90% wheat accessions (2)	Heterozygous in no more than 10% wheat accessions (3)	Minor allele frequency > 5% (4)	Final % of initial
Markers used for MAS*	65	57	39	35	11	**16.9**
Previously associated with traits in GWAS studies using iSelect 90 K array	4,537	1,160	774	626	299	**6.6**
Found in wheat genes deposited to GenBank (NCBI) database**	3,500	1,045	603	535	128	**3.7**
Confer missense or nonsense mutations	26,149	9,276	5,080	3,685	2,288	**8.7**
Located within gaps between others and are highly polymorphic	25,750	4,957	2,979	2,597	730	**2.8**
Total	***60***,***001***	***16***,***495***	***9***,***475***	***7***,***478***	***3***,***456***	***5.8***

* MAS – marker-assisted selection. ** Not as parts of large contigs or whole chromosomes

**Fig. 1:**
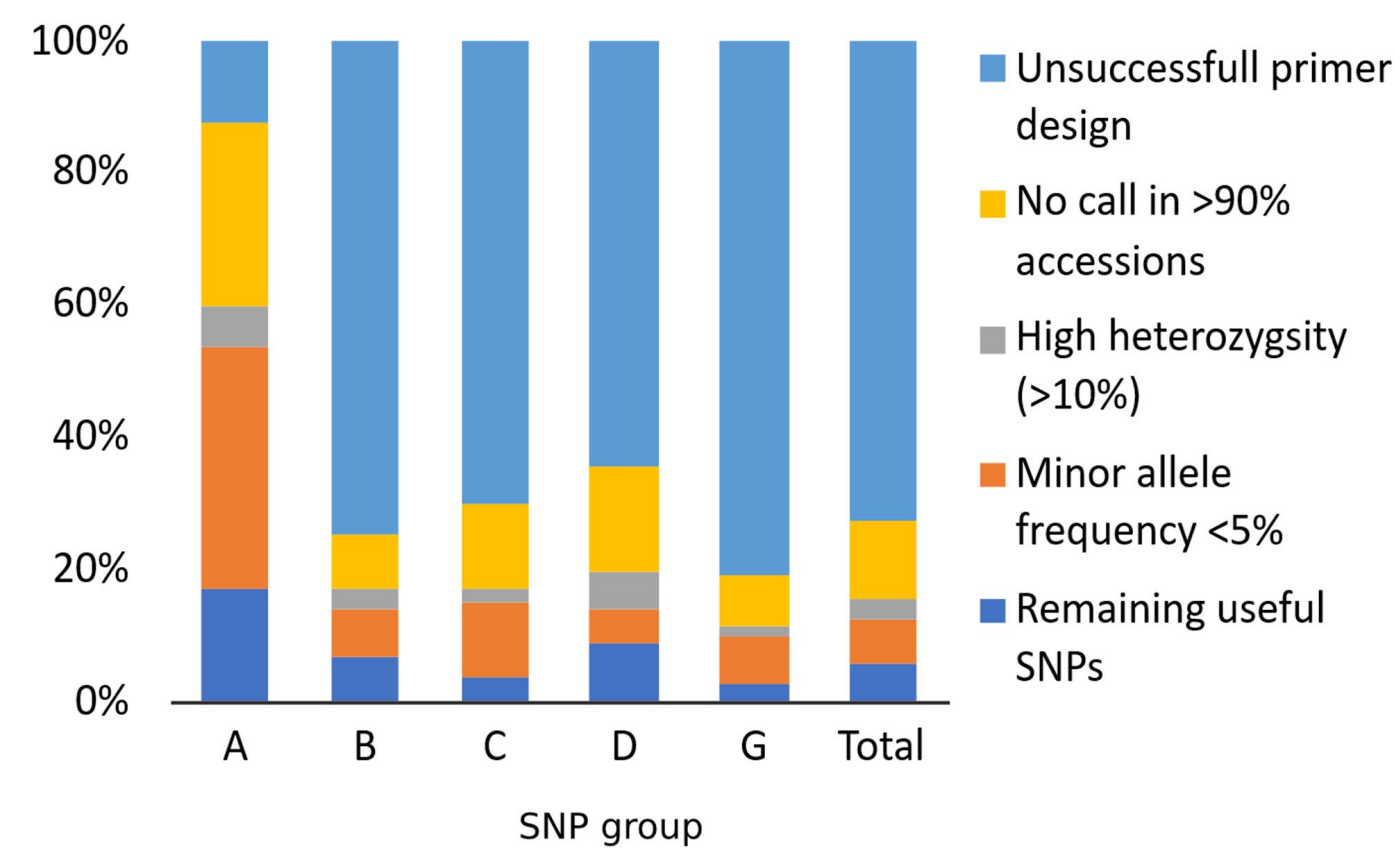
Reasons for removing SNPs from the initial 60 K set. The SNP groups are as follows: **A** based on KASP MAS markers, **B** from previous GWAS that use iSelect-90 K array, **C** mutations in genes which sequences of were deposited to NCBI GenBank database, **D** protein-changing mutations, **G** random SNPs for gap filling

The key step of targeted genotyping by sequencing is amplification of DNA fragments containing the desired SNPs. More than 70% of the initially selected 60 K SNPs were excluded due to unsuccessful automatic oligonucleotide probe design. This problem was least pronounced for those SNPs for which KASP markers had previously been designed (Fig. 1). Totally, for 16.5 thousand SNPs specific enrichment probes were successfully created (Table 1). The results of genotyping of winter wheat collection using this 16.5 thousand SNP set are available in Additional file 6.

The second reason that led to rejecting SNPs from initial set was unsuccessful calling in the majority of wheat accessions. This problem is more frequent for mutations in protein-coding regions (Fig. 1). That may be caused by high GC content of SNP-surrounding sequences or by the presence of chromosome deletions or translocations. Hybridization of probes to GC-rich sequences and polymerase reaction over them could be challenging because the target DNA may form thermo-stable structures resulting from incomplete denaturation, reannealing, and self-annealing [51]. And protein-coding regions are known to be more GC-rich than random genome fragments [52]. Totally, about 9.5 K SNPs detectable in more than 90% accessions were obtained (Table 1).

**Fig. 2:**
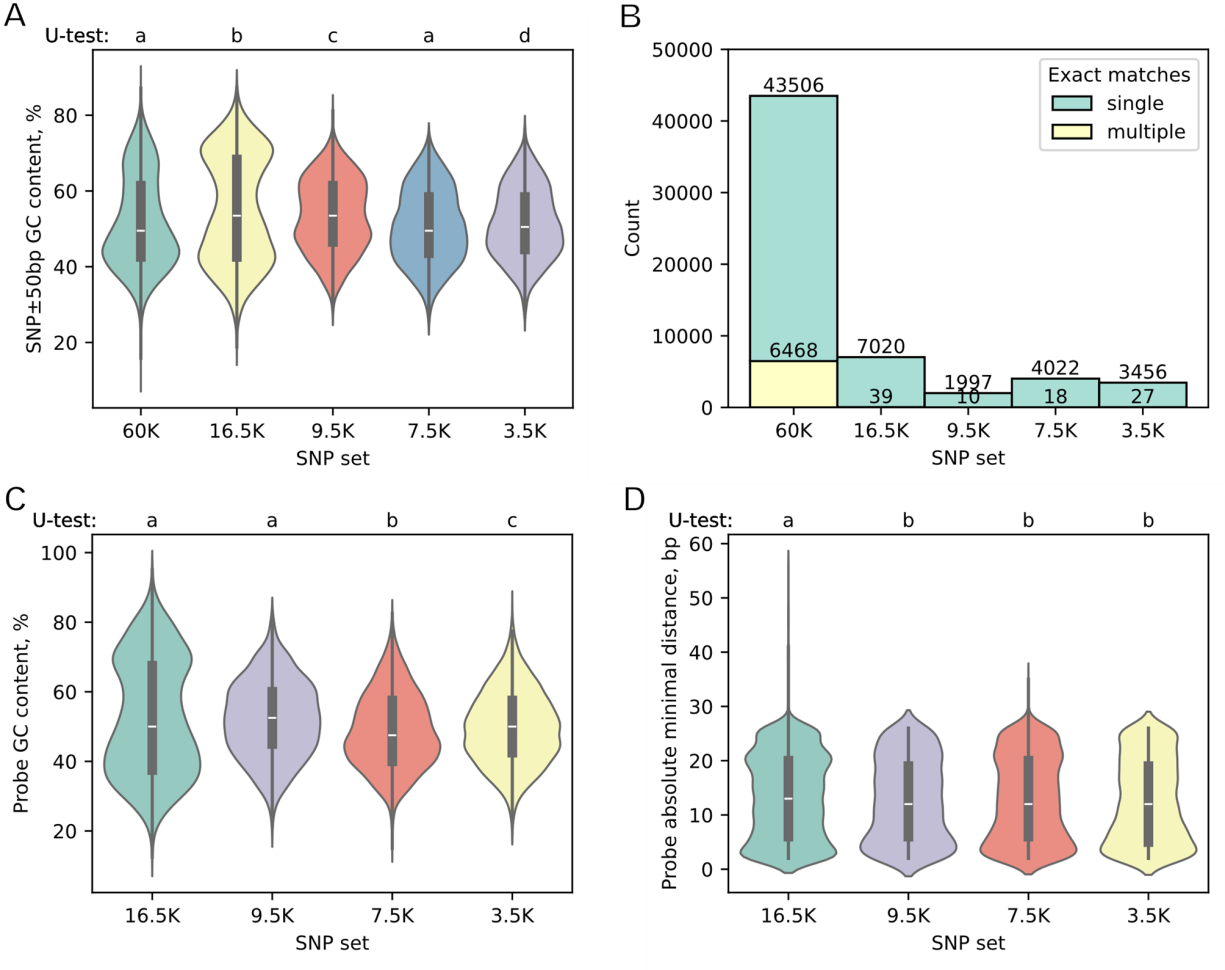
Diagrams showing the properties of sequences surrounding SNPs in a range of ± 50 bp (**a**, **b**) and properties of enrichment probes (**c**, **d**) in different steps of SNP filtering. Each subset on diagram includes only those SNPs that were left in that very group and did not pass to the next smaller subset (if such present), 60 K is an initial set, and 3.5 K is a final subset. Diagrams (**a**) and (**c**) represent the percent of G and C nucleotide bases in SNP ± 50 bp fragment and enrichment probes for these SNPs, diagram (**b**) represents the number of SNP ± 50 bp fragment exact matches in genome (in categories single or multiple), and diagram (**d**) shows the minimal distance between the probe 3′-end and an SNP (for an SNP a single specific probe or two probes if possible were developed). On the violin plots the letters above diagrams represent homogenous groups according to Mann-Whitney U Test

To understand the reasons why most of the initially suggested SNPs were excluded from the final set, we examined the properties of the adjacent sequences around them (Fig. 2). We calculated the proportion of GC nucleotide base pairs within the ± 50-bp range and mapped these fragments to the latest version of the wheat Chinese Spring genome assembly, RefSeq2.1.

About 10% of SNPs in the initial set have adjacent sequences that exactly match more than one locus in the genome (Fig. 2B). This could be due to the polyploid nature of bread wheat (*Triticum aestivum* L., 2n = 6x, genomic formula BBAADD) and the similarity between the sequences of the A, B and D subgenomes. It is impossible to select specific oligonucleotide probes for these SNPs.

There is a significant change in the distribution of GC content during SNP filtration. The initial 60 K set has a wide range of adjacent sequence GC contents, with most SNPs having %GC values below 50 (Fig. 2A). Selecting SNPs that can be (theoretically) targeted with specific probes leads to a much higher proportion of adjacent sequences with high GC contents in the 16.5 K set. The distribution of GC content in probes is generally similar to that in the surrounding sequences (Fig. 2C). Further testing of the probes in an experiment showed that only those with a narrower range of GC contents successfully provided genotyping data. Therefore, 95% of the 3.5k probes have a GC content between 33% and 70%, with an average value of around 50%.

A few SNPs were not successfully genotyped because the probe was somehow selected too far away from the SNP, and most successful probes had a distance of their 3ʹ end ranging from 5 to 25 bp away from the target SNP (Fig. 2D).

**Fig. 3:**
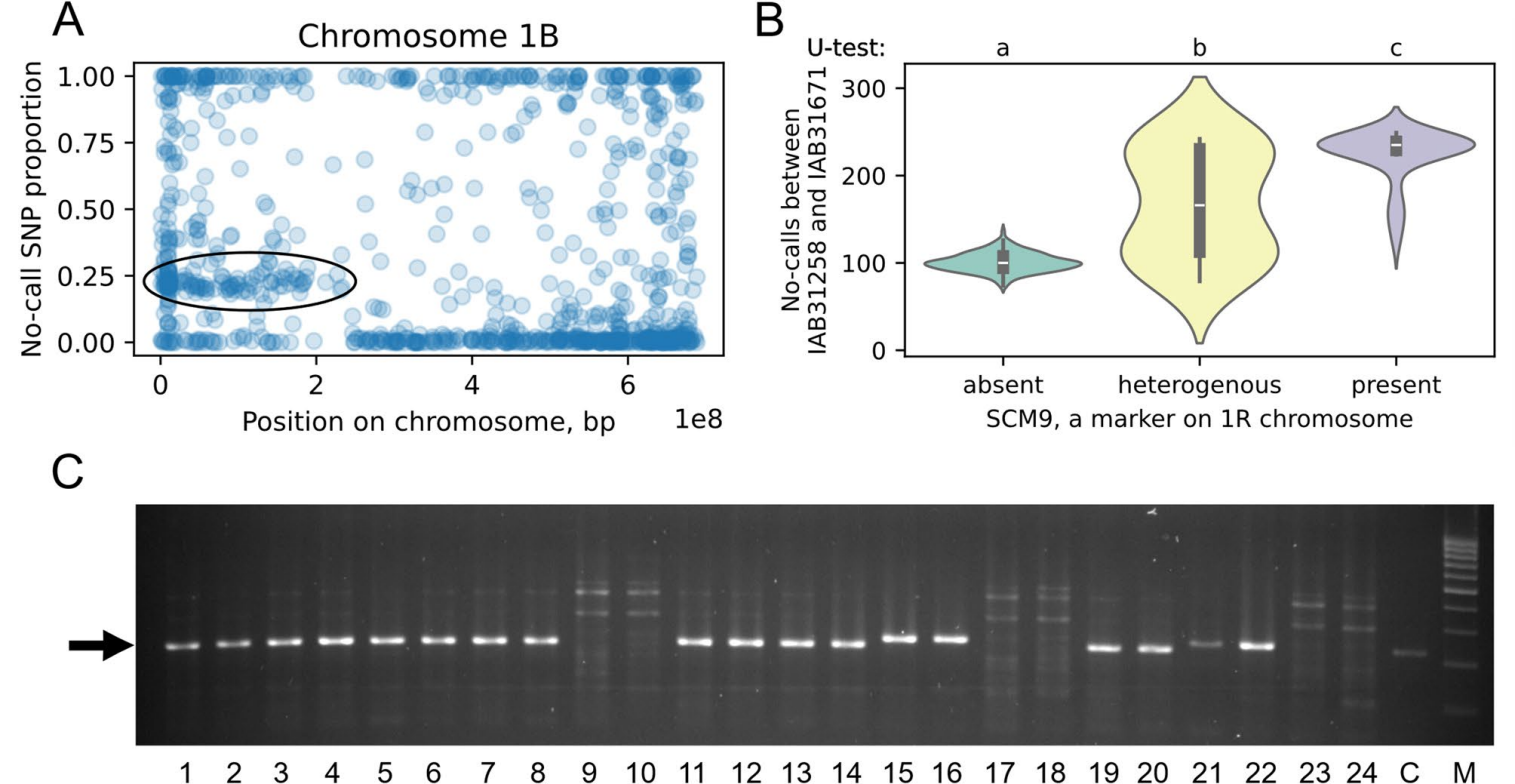
Proportion of missing (no-call) values for SNPs of the 16.5 K set mapped on chromosome 1B pseudomolecule of RefSeq1.0 wheat genome assembly. The circled area shows unusually high rate of missing values on the short chromosome arm, which may result from frequent occurrence of 1RS.1BL wheat-rye translocation in wheat collection (**a**). The number of missing values between markers IAB31258 (chr1B: 364,285) and IAB31671 (chr1B: 155,411,045), in connection with 1BL.1RS translocation, detected by SCM9 microsatellite marker located on 1RS chromosome arm (**b**). An example of agarose gel electrophoresis of SCM9 marker. The characteristic DNA fragment is shown by arrow. C – is positive control (PCR with DNA of rye). The last line M is a 100–1000 bp DNA ladder M100 (Syntol JSC, Moscow, Russia) (**c**)

In our study, many wheat lines got missing values of SNP-seq markers on the short arm of chromosome 1B (Fig. 3). We assumed, that this could be a sign of frequent occurrence of 1RS.1BL wheat-rye translocation among the lines. Using missing values number between markers IAB31258 (chr1B: 364,285) and IAB31671 (chr1B: 155,411,045) we predicted that 39 lines with a large number of missing values probably carry the 1RS.1BL translocation, while other 152 do not. Also, there were 9 lines with an intermediate number of missing values, which are difficult to assign to one of the groups. The prediction of the presence of 1RS.1BL translocation showed good correlation with PCR-amplification of SCM9 microsatellite marker specific for the 1R rye chromosome (Fig. 3B-C; Additional file 7). Many other chromosomes, including 1 A, 2 A, 2B, 3 A, 6B and 7B, also possess similar but less pronounced signs of chromosome rearrangements, like deletions or alien translocations (Additional file 1, Supplementary Fig. 1).

Heterozygosity, that may be interpreted here as heterogeneity existing within an accession, when it is high enough for a single SNP, may indicate that genetic information was obtained simultaneously from several similar loci due to erroneous mapping of DNA reads. Accordingly, we rejected some SNPs due to they were detected in heterozygous state in more than 10% wheat accessions, thus about 7.5 K polymorphisms were remaining on this step of filtering (Table 1).

The last filtering step removed SNPs which possess a minor allele frequency below 5%. That was made, as too low number of genotypes in a group for comparison may result in erroneous conclusions in GWAS analysis. SNPs selected based on MAS markers primers and those found in GenBank-deposited sequences have been significantly reduced in number by this filter. For MAS SNPs we concluded that this reduction results from a limited genetic diversity of the collection, where some alleles are absent or rare (Additional file 8). The most significant reduction in this step occurred in the D subgenome (Additional file 1, Supplementary Fig. 2), which reflects its limited genetic diversity in wheat. Finally, about 3.5 K markers were selected for further analysis (Table 1).

### Breeding markers diversity

The SNPs selected based on breeding MAS markers and detected by targeted sequencing were specifically checked for their diversity (Additional file 8). Expectedly, no or limited diversity was found for *Vernalization* gene markers, as most of accessions in a collection are winter forms, while only Afina, Karavan and Velena are facultative. Accordingly, these three accessions possess a rare in our collection variant of IAB10571, a marker of *VRN-D1* gene, while winter forms show ‘no-call’ variant of that marker. No diversity in our collection was found for the markers of *Grain Width and Weight 2* (*TaGW2-A1*) gene, *Waxy* (*Wx-D1a*) gene connected with amylose-amylopectin ration in starch, grain protein content gene *Gpc-B1*, and grain yellow pigment gene *Psy1-D1.* For genes like yellow rust resistance gene Yr15, earliness per se gene associated with marker CIMwMAS0123, photoperiod genes *Ppd-A1* and *Ppd-B1*, SUCROSE SYNTHASE gene associated with thousand grain weight *(Sus2-2B)*, endosperm hardness gene *Pina-D1*, cereal cyst nematode resistance gene *Cre8*, and leaf rust resistance gene *Lr47* the alternative alleles were rare in our collection, and we revealed accessions carrying these alternative alleles (Additional file 8).

As only a fraction of initial number of MAS-markers were successfully converted to informative polymorphisms detected by targeted genotyping by sequencing, and these markers are the most interesting from breeders’ point of view, we decided to complement the obtained genotypes data with information obtained using conventional PCR markers. The polymorphisms for which the genotype data were complemented are represented in Table 2.

**Table 2:** Additional polymorphisms detected by PCR markers included in the genotype dataset

Polymorphism	Gene	Designation of alleles in dataset	Marker reference
IAB04062	*PPD-D1 (Photoperiod D1)*	T – 288 bp, *Ppd-D1a*, photoperiod-insensitive; C – 414 bp, *Ppd-D1b*, photoperiod-sensitive*	[37]
IAB07969	*RHT-D1 (Reduced height D1)*	G – *Rht-D1a*,* tall;* T – *Rht-D1b*, semidwarf	[35]
IAB07477	*RHT-B1 (Reduced height B1)*	C – *Rht-B1a*,* tall;* T – *Rht-B1b*,* semidwarf*	[35]
IAB60001	*RHT-B1*	A – *Rht-B1a*,* tall;* T – *Rht-B1e*,* semidwarf*	[36]

* For this marker for the simplicity of data processing the indel was coded as dummy SNP in the same position of reference genome

### Distribution of markers on chromosomes

**Fig. 4:**
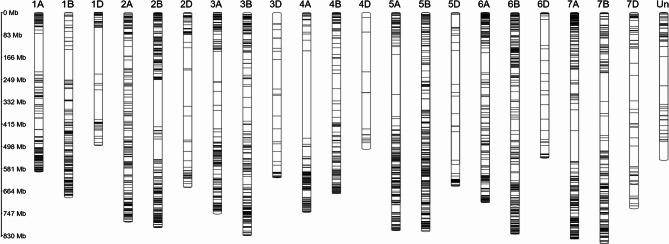
A map of 3,456 confidently detectable and polymorphic SNP markers on wheat chromosome pseudo-molecules (Chinese Spring RefSeq 1.0 genome assembly) that were used for population structure inference and GWAS in this study. Each marker is represented by a thin black horizontal line drawn across the chromosome. The scale on the left indicates distances in mega base pairs. The pseudo-molecule designated as ‘Un’ is a collection of unmapped contigs

The final marker set suitable for conducting population structure and GWAS research consisted of 3,456 SNPs providing coverage of all the common wheat chromosomes. However, the coverage was uneven. Most of the markers occurred at distal parts of the chromosome arms, while the middle parts of chromosomes, assumably corresponding to pericentromeric parts, were lacking polymorphic molecular markers (Fig. 4). Uneven distribution of markers corresponds to an uneven distribution of genes, which are known to occupy mainly the distal chromosome regions [53].

### Population structure

**Fig. 5:**
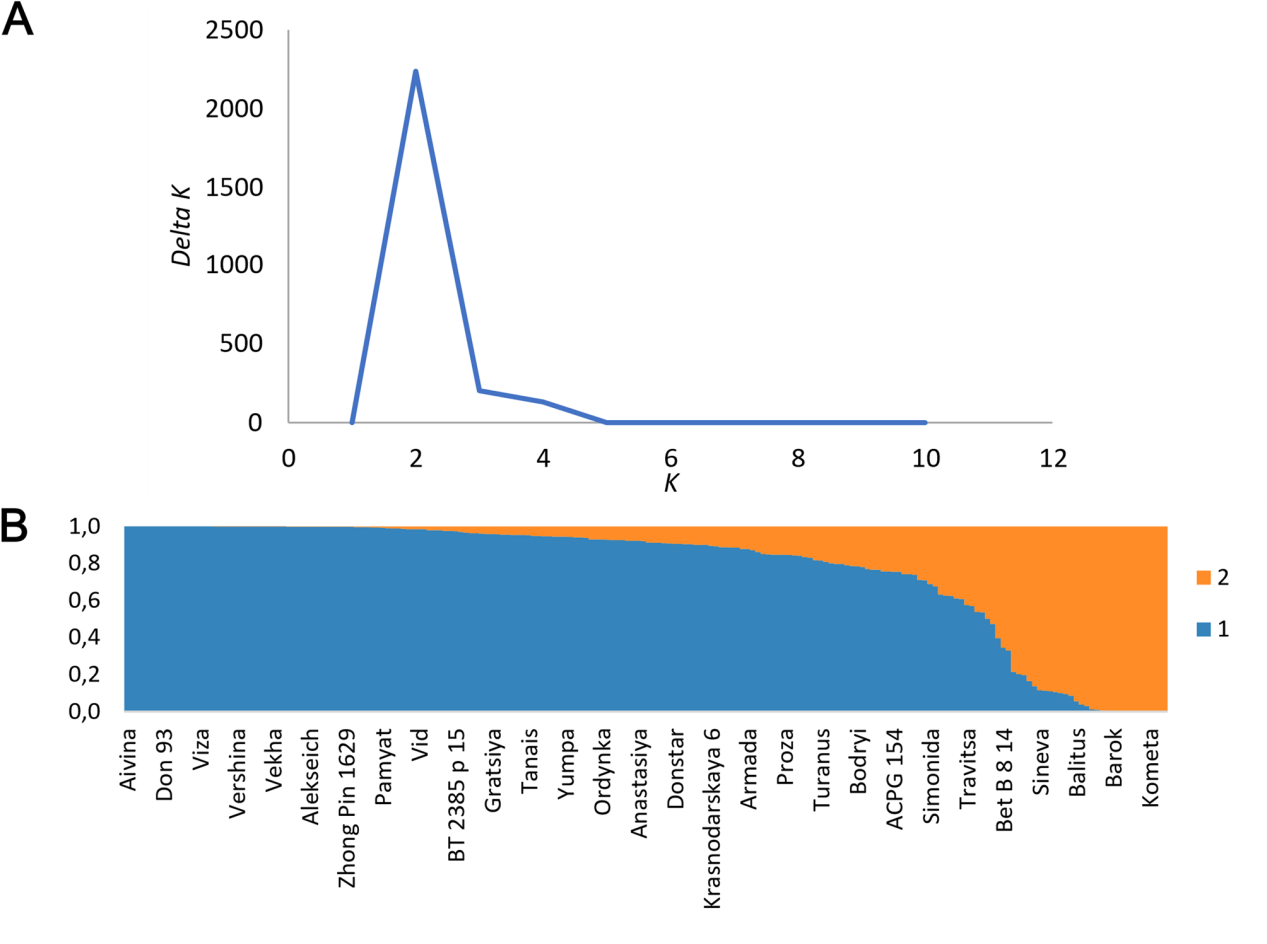
**a** The graph of the *∆K = M(|L’‘(K)|)/sd(L(K))* value. **b** Population structure diagram for K = 2 inferred subpopulations

Analysis of the population structure using STRUCTURE 2.3.4 software for 200 wheat accessions genotyped by 3,458 selected SNP markers showed that the most probable number of subpopulations is 2 (Fig. 5a). That corresponds to the maximum of ΔK, which is the second derivative of the natural logarithm of the likelihood of the model with respect to the number of subpopulations, in relation to its standard deviation, calculated using several pseudo-samples [43]. The diagram of the population structure with an assumption of two subpopulations is shown in Fig. 5b.

**Fig. 6:**
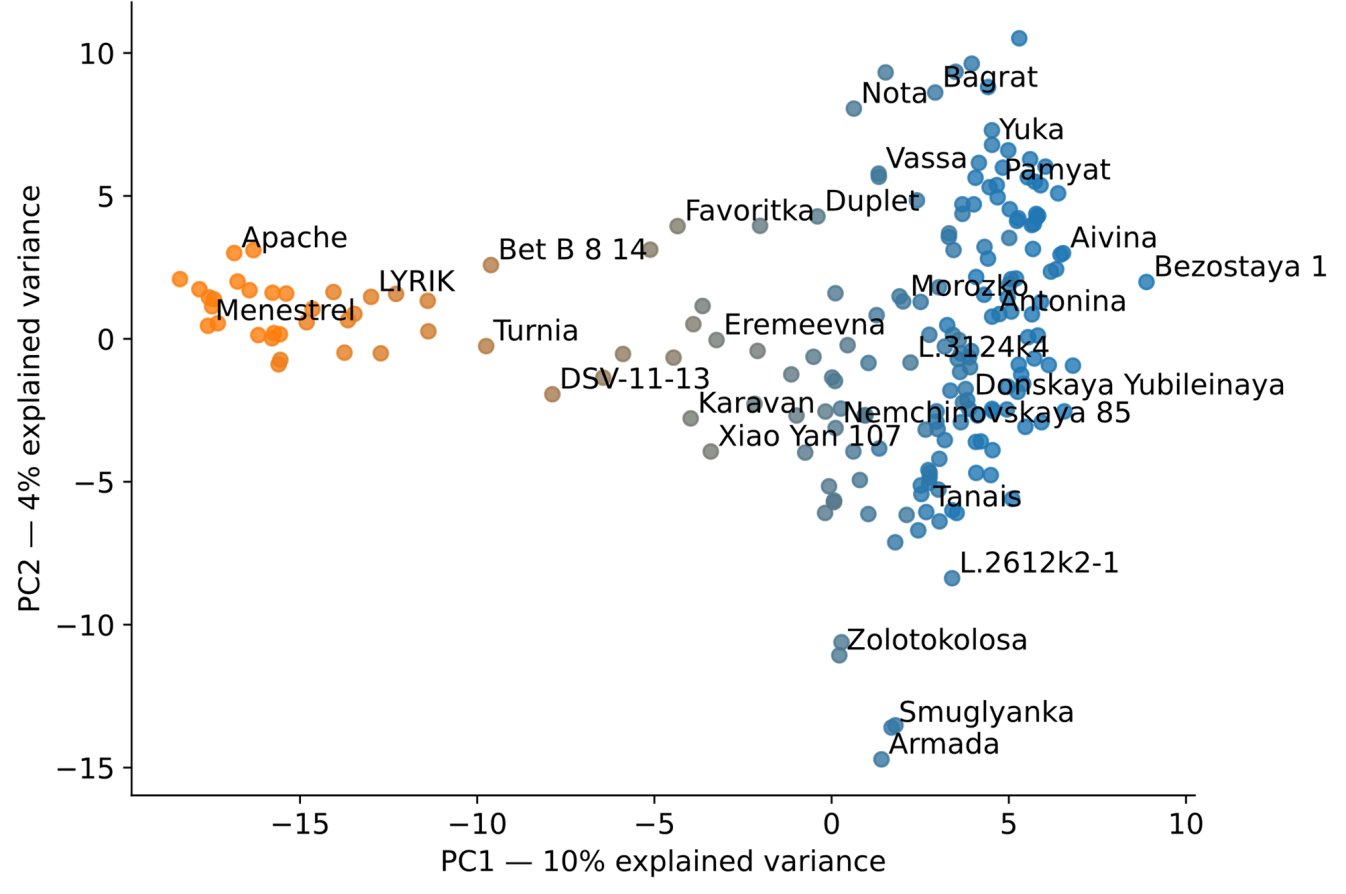
Analysis of the population structure by the principal component method. The color shows the subpopulations identity inferred by the STRUCTURE 2.3.4 software. The dots show individual wheat lines in the coordinates of the first (PC1) and second (PC2) principal components

The principal component method is also usually applied to account for population structure based on genotyping data [54]. The representatives of different subpopulations usually form clusters in the space of the first principal components. In our case, plotting the wheat accessions in the space of the first two principal components of genotypes also resulted in two clusters (Fig. 6). These clusters correspond to subpopulations 1 and 2, identified using STRUCTURE 2.3.4 software.

A smaller cluster of accessions consists mainly of European varieties originating from France, Germany, Austria, Poland, Czech Republic, Bulgaria and UK. A larger and more diverse cluster, as should be expected, is a group of Russian cultivars. Within each of two clusters, the separation by the region of origin is not pronounced. Also, there is a “bridge” between two clusters connecting them – a group of cultivars probably created by hybridization of geographically distant accessions, consisting of both domestic and foreign cultivars. There are also some accessions found within each of the two groups that seem not to belong to them by geographical origin. This fact suggests that a small part of the wheat cultivars both in Russia and abroad could have been created by hybridization between the parent forms, each of which has a remote geographical origin relative to the area in which the breeding takes place. At the same time, most of the cultivars appear to be created by hybridization within the local gene pools (Additional file 1, Supplementary Figs. 3 and 4).

**Fig. 7:**
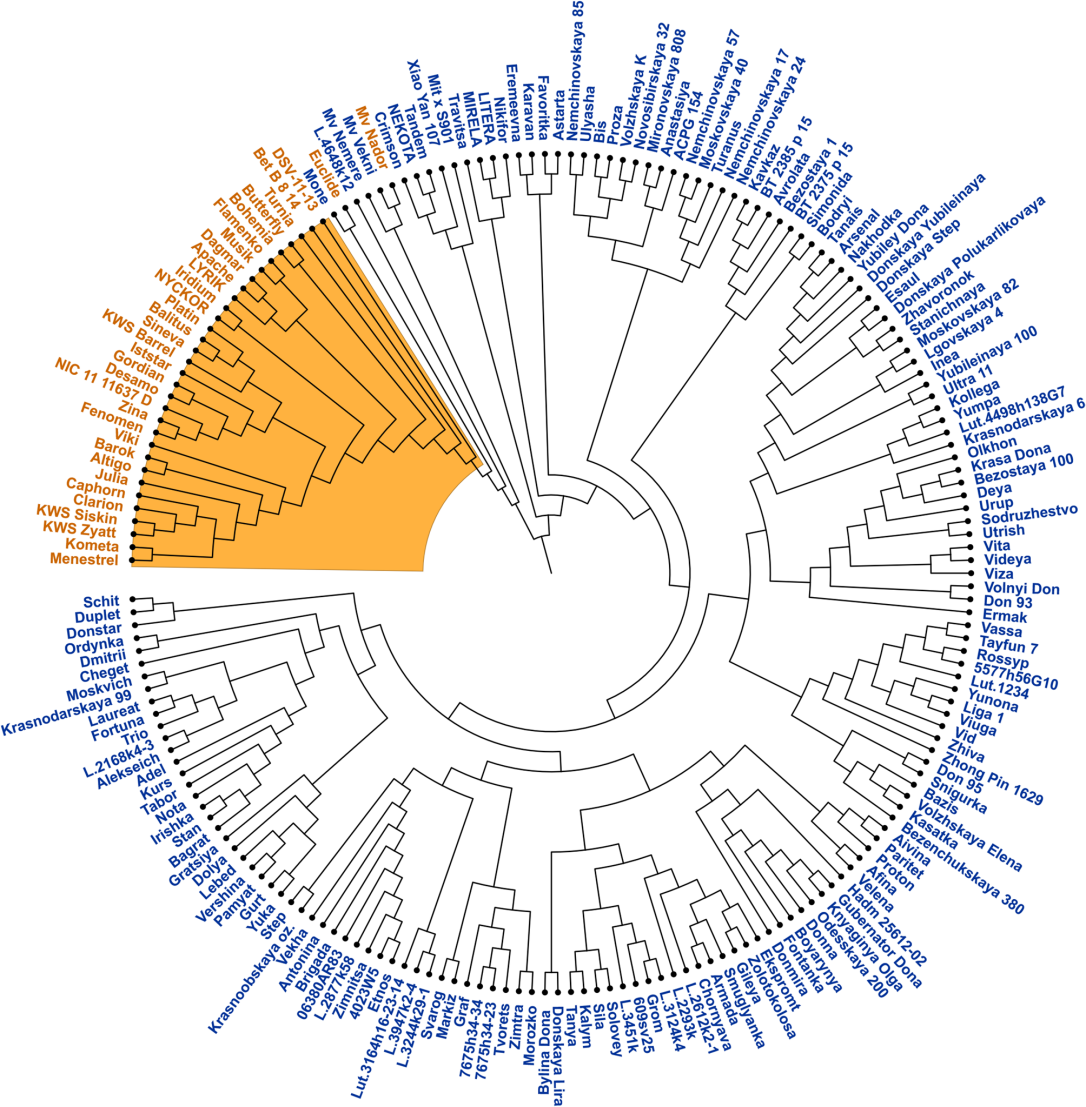
The neighbor-joining tree drawn based on an identity-by-state distance matrix for 200 wheat cultivars and breeding lines using 3,456 polymorphic markers, with the root at midpoint. The accession labels are colored according to groups revealed by population structure analysis

Also, based on polymorphic markers, a molecular phylogenetic dendrogram of wheat line similarity was constructed (Fig. 7).

This neighbor-joining tree confirms the results of other clustering approaches, at the same time providing the information on the pairs of the most similar wheat cultivars and lines (Fig. 7). The same tree, with indicated locations of wheat accessions’ origins, can be seen in Additional file 1, Supplementary Fig. 4.

### Marker-trait associations

**Fig. 8:**
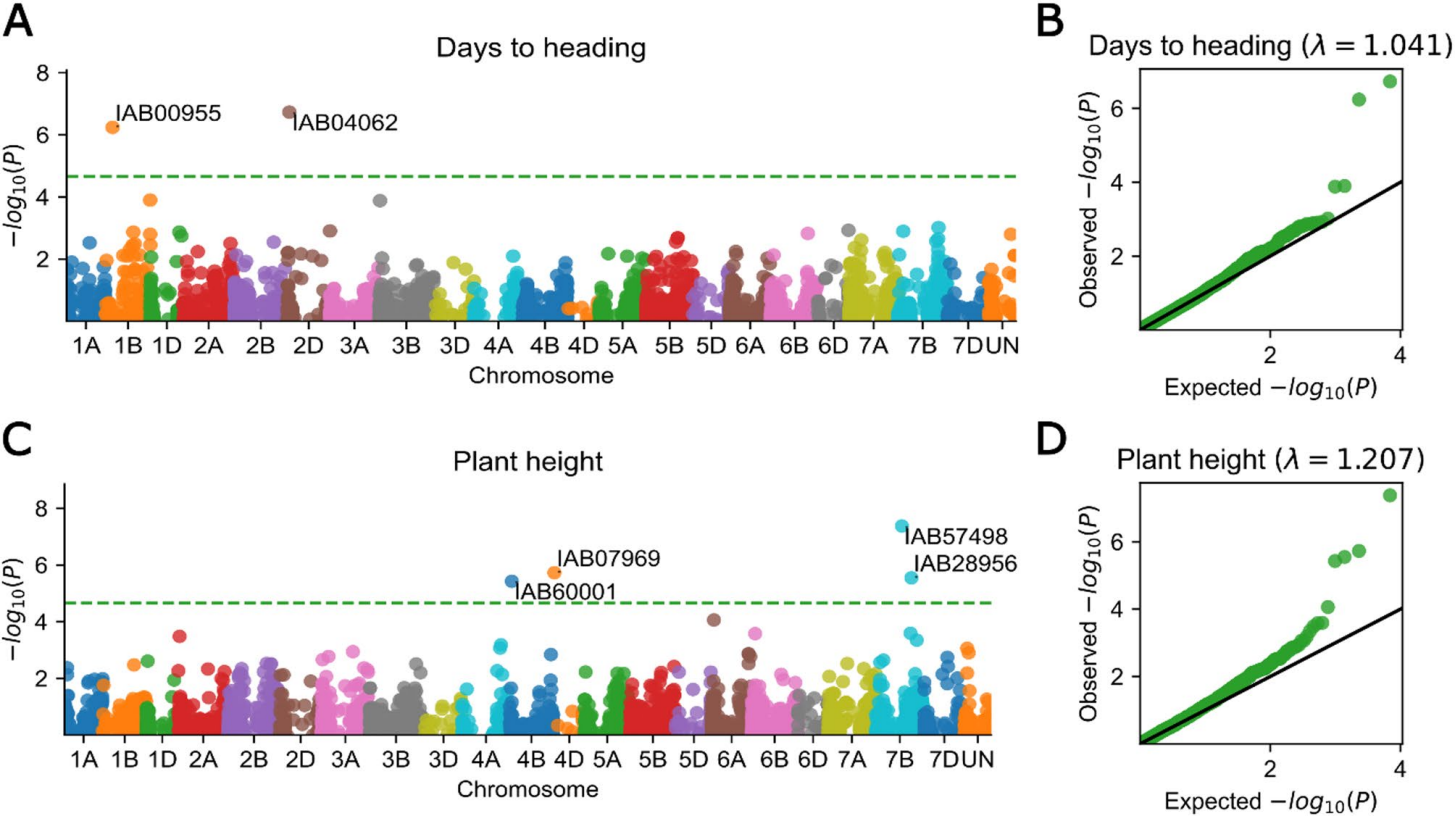
Examples of Manhattan-plots (**a**, **c**) and QQ-plots (**b**, **d**) for showing significant marker-trait associations observed in our study for the 3-year BLUES (best linear unbiased estimations) of time interval from sowing to heading (**a**, **b**) and plant height (**c**, **d**) in a collection of 200 winter wheat accessions genotyped with 3,456 SNP-seq polymorphic markers supplemented with PCR markers for *RHT-B1* (IAB60001 – *Rht-B1e*), *RHT-D1* (IAB07969 – *Rht-D1b*) and *PPD-D1* (IAB04062 – *Ppd-D1a*) genes. Manhattan-plots and QQ-plots for other traits could be found in supplementary materials

**Fig. 9:**
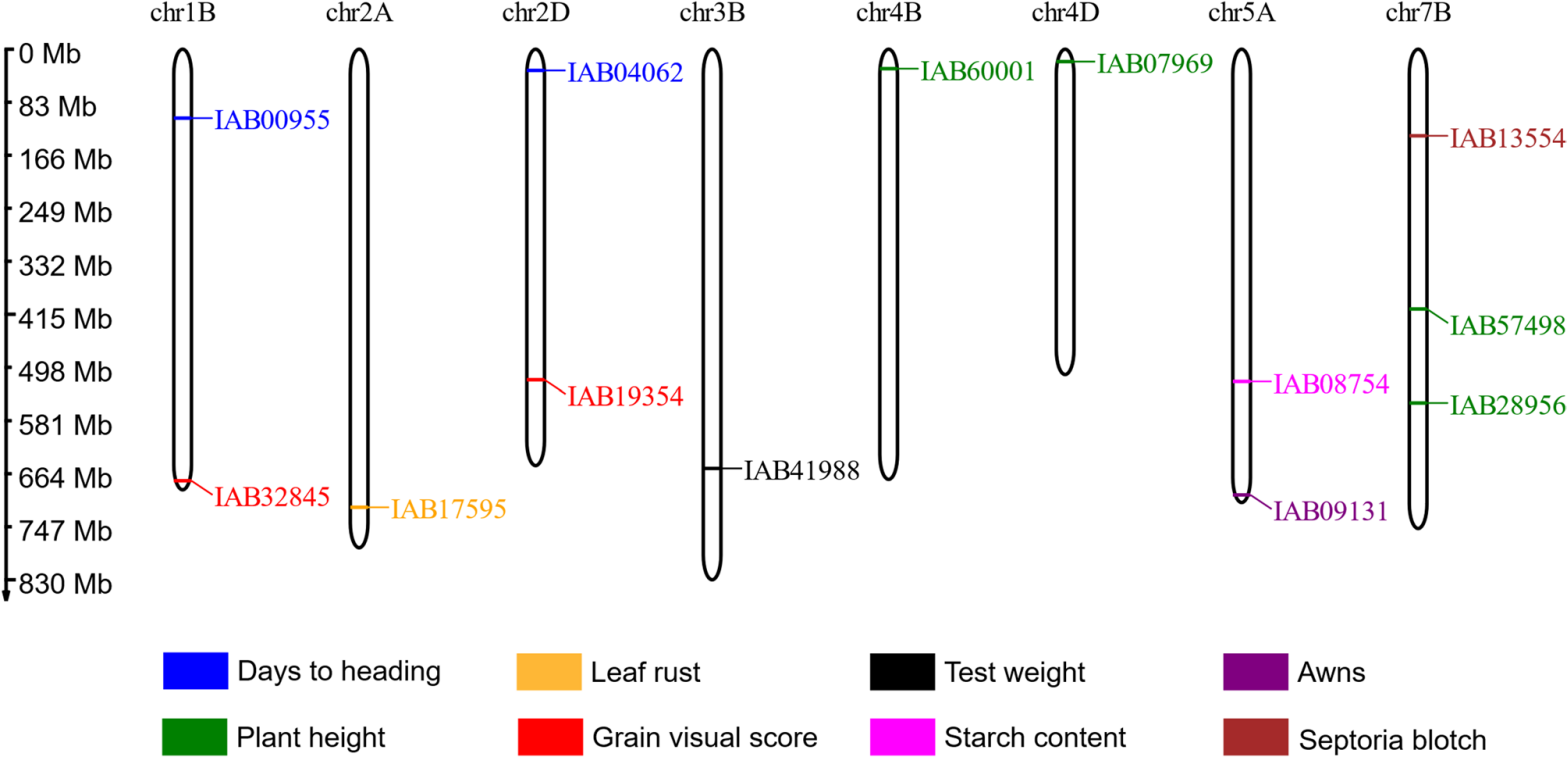
Physical map of the markers for which significant marker-trait associations were found. The color of marker label designates the trait: awns — purple (IAB09131), plant height — green (IAB60001, IAB07969, IAB57498, IAB28956), days to heading— blue (IAB04062, IAB00955), grain visual score — red (IAB19354, IAB32845), test weight — black (IAB41988), starch content — magenta (IAB08754), leaf rust — orange (IAB17595), septoria blotch on flag leaf in 2019 — brown (IAB13554)

Based on mixed linear models (MLM) analysis, significant marker-trait associations (MTAs) were observed for 3-year BLUES of days to heading on chromosomes 2D and 1B, for plant height on chromosomes 4B, 4D and 7B (Fig. 8), for 1000 kernel weight on chromosome 2D, for grain visual score on chromosomes 2D and 1B, for leaf rust resistance on chromosome 2 A (Fig. 9; Additional file 1, Supplementary Fig. 5; Additional file 9). The presence of awns, as a constant characteristic of accession, was associated with a marker on chromosome 5 A, and that was the most significant association of all we found. Septoria blotch damage of the flag leaf was associated with a marker on chromosome 7B for single-year data in 2019. Near-to significant association by p-value (but could also be considered as significant by QQ-plot) was found between grain yield (t/ha) and a marker of *PPD-D1* gene of photoperiod sensitivity. Also, a near-to significant association could be found for test weight and a marker on chromosome 3B; starch content and a marker on chromosome 5 A. For other traits, including grain protein content, gluten content, lodging resistance, and stripe rust resistance no significant marker-trait associations were observed. The plots showing a direct connection of plant phenotypes with SNP genotypes are shown on the Supplementary Fig. 6, Additional file 1.

**Fig. 10:**
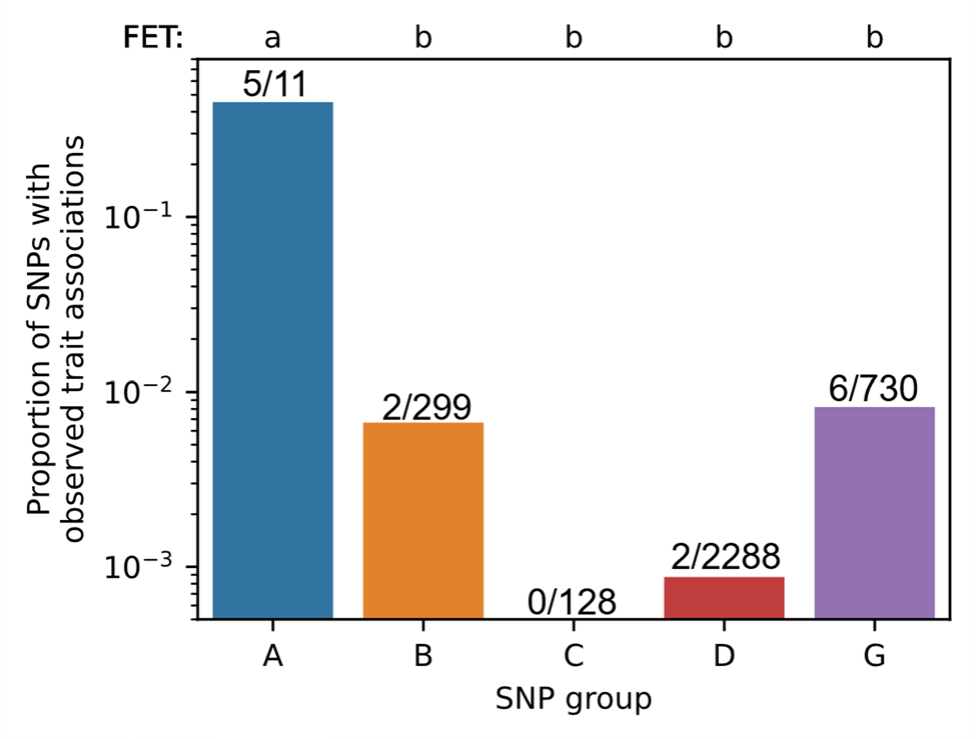
Proportion of SNPs of the final 3.5 K set for which significant marker-trait associations were found. The SNP groups are as follows: **A** based on KASP MAS markers, **B** from previous GWAS that use iSelect-90 K array, **C** mutations in genes which sequences of were deposited to NCBI GenBank database, **D** protein-changing mutations, **G** random SNPs for gap filling. Letters above the diagram designate homogenous groups of non-significantly different values according to Fishers exact test (FET)

At the beginning of the study, we grouped SNPs according to the information source we used to find them. Looking at the success of finding marker-trait associations (MTAs) for these different SNP groups (Fig. 10), we can say, that the most successful was the group of SNPs derived from KASP markers that are widely used in breeding. The SNPs from other sources were far less successful in finding MTAs in our study, and were not statistically different in that success from each other. The SNPs from previous GWAS produced in our study the same frequency of significant MTAs (with any traits), as random polymorphic SNPs that were used to cover the large gaps between markers in the initial set. Interestingly, SNPs from protein-coding genes that should cause amino-acid sequence change produced no more, and even less MTAs than random SNPs, however we cannot state this with statistical confidence (Fig. 10b).

Seven SNPs of the 13 that were significantly associated with certain traits in our study, were not derived from and are not overlapped with any known markers annotated to the wheat genome RefSeq1.1. This annotation includes Axiom 820 K, TaBW280K, Infinium 90 K and 9 K, as well as DArT array markers (see Additional file 9). Therefore, markers for these seven SNPs can be considered novel.

### KASP markers for valuable SNPs

For SNP-seq markers that showed significant associations with the agronomical traits, we designed KASP markers (Additional file 1, Supplementary Table 2) to validate sequencing data and to provide breeders with useful tools for marker-assisted selection. We tested these KASP markers on 15 winter wheat varieties selected based on known SNP-seq genotypes. The KASP markers demonstrated good polymorphism discrimination (Additional file 1, Supplementary Fig. 7). The results of KASP genotyping completely coincided with the sequencing data in 15 wheat accessions, with the only exception of marker IAB00955 which showed 93% accuracy (1 mismatched result out of 15, Additional file 1, Supplementary Table 3).

## Discussion

### Efficiency and prospects of SNP-seq method

Targeted genotyping by sequencing of single nucleotide polymorphisms (SNP-seq) is a new high-performance genotyping method. Compared to other methods, SNP-seq has a lower cost of genotyping while allowing researchers to independently create sets of markers and obtain high-quality data. A previous study that applied SNP-Seq technology to wheat noted that targeted GBS can aid in making a bridge between data obtained using genotyping arrays and data obtained through sequencing-based genotyping technologies. Also, sequencing technology’s advantage is the ability to identify non-target mutations if they are present near targeted loci, as well as the opportunity to identify not just biallelic but also triallelic SNPs [17].

We initially took 60 thousand SNPs. For 16.5 thousand of these, enrichment probes were designed. Finally, approximately 3.5 thousand were found to be high quality and suitable for use in further studies. Despite drastic SNP dropout from the initial set, the 16 K panel of markers developed for this study showed comparable efficiency to the Illumina iSelect 90 K DNA array, which usually gives only 7 to 20 thousand SNPs of high quality for a given study [1, 55–58]. The advantage of SNP-seq technology is the possibility of easy optimization of the marker set, which may significantly reduce the cost genotyping in the following studies. Additionally, it is possible to use an even smaller number of markers for certain crosses between the studied lines if we need to perform a quantitative trait loci (QTL) mapping study or perform genomic selection.

The main reasons for the drastic loss of SNPs during panel design were the non-optimal GC content around target SNPs and the complexity of the wheat genome due to its allopolyploid nature. Common wheat is an allohexaploid species, meaning that almost any gene has three more or less similar copies in subgenomes B, A and D. If a target SNP is located in a conserved region of a gene, it can be difficult to find suitable differences between homoeologues for precise targeting. Partial unsuccess in genotyping experiment was assumably due to non-optimal probe GC content, which should be in a range of 30 and 70%, aiming at 50%. Thus, for targeted seq-SNP experiments a significant limitation exists, due to that not every single SNP could be targeted by enrichment probes, which should have GC content in a narrow range, and should have a single hybridization locus all over genome, which could be a problem for polyploid plant species. Similar reasons for enrichment probe design failure were suggested in previous study [17].

### Failed SNP calls may carry important information

Earlier studies have shown that SNPs with failed allele calls on genotyping arrays may contain important information about genomic structural variants, such as deletions, and these SNPs can be highly predictive of important agronomic traits [59]. In our work, we did not use missing SNP values for trait prediction. However, we have successfully demonstrated that missing SNP calls can be used to predict alien translocations, in our case, an 1RS.1BL wheat-rye translocation, which is the replacement of the short arm of chromosome 1B with the short arm of chromosome 1R from rye, which carries a number of important disease resistance genes – Lr26, Sr31, Yr9 and Pm8 [60]. Translocation 1RS.1BL is the most widespread alien translocation in wheat [18]. It is known to increase the aboveground biomass and yield potential of plants, as well as improve root system growth and drought resistance. However, it does reduce baking qualities due to changes in the composition of grain proteins caused by the introduction of *Secalin-1* (*Sec-1*) and the loss of the *Glutenin-B3* (*Glu-B3*) locus [61, 62].

Translocation 1RS.1BL is not the only used in wheat breeding. Other wide-spread wheat alien translocation is the T6VS.6AL with a chromosome fragment from *Dasypyrum villosum* carrying powdery mildew resistance gene *Pm21* [63]. Numerous other alien translocations carrying resistance genes were identified in wheat [64]. Non-alien translocations are also common in wheat genome, however, only some of them are non-reciprocal, for example T2A:1D, T7A:5B and T6A:4B, and may result in loss or double-copy of some genetic material [65]. Failed allele calls obtained from SNP-seq or other high-throughput genotyping methods can be used to identify these various translocations in wheat breeding material. However, this topic deserves a separate study.

### No-diversity SNPs also may provide important information

Markers with no diversity or rare alternative alleles are usually considered useless for association studies, but they still may contain important information about the genetic diversity of a population and provide clues for crop improvement. For example, our collection of winter wheat did not contain any variation in the *TaGW2-A1* and *Gpc-B1* genes, which means that introduction of donors of mutation variants of these genes in a breeding program could lead to significant improvements in grain size and grain protein content. Accessions with rare but favorable alleles should also be used more frequently in crosses to produce cultivars with improved agricultural traits. Another example is the marker for the *Vrn-D1* gene, which had a high rate of no-call variant and a rare alternative variant. Manual screening of genotyping results of *Vrn-D1* gene marker showed a connection between the presence of a rare alternative variant and the facultative growth habit of wheat. Our findings are supported by previous studies that have shown an association between the *Vrn-D1* gene variants and a facultative growth habit [66].

### Insights from genotype clustering

The different methods of clustering applied to the genotypic data yielded similar results – the winter wheat collection is divided into two subpopulations. One of these subpopulations (the larger one) consists primarily of Russian cultivars, while the other (the smaller one) contains mostly foreign European cultivars. Therefore, the genetic differentiation between the subpopulations can be attributed to their geographical origin. Analysis of the positions of the accessions in the coordinate system of the first two principal components suggests that most new cultivars were created by hybridization within the local gene pool. Rarely, they were developed by crossing local cultivars with foreign ones, and even more rarely, by using completely foreign germplasm in breeding.

### Marker-trait associations and candidate genes

#### Presence of awns

Presence of awns, the hair-like structures growing from lemmas in inflorescences of grasses, is a stable morphological characteristic of wheat cultivars. Variation in awn length in domesticated wheat is controlled by three major genes, and most commonly associated with variation at the awn suppressor locus *Tipped1-B1*. In our study we found association between the presence of awns and IAB09131 SNP marker on chromosome 5 A (chr5A: 698,510,016). This SNP is located within the TraesCS5A02G542600 gene coding a hexose transporter, and in 18.5 kilobase pair from the C2H2 zinc finger transcriptional repressor gene TraesCS5A02G542800, which was previously identified as causal for awn growth regulation in *Tipped1* locus [67]. Interestingly, that SNP on chromosome 5 A (RAC875_c8642_231) which was associated with presence of awns in our study, was earlier associated with calcium accumulation in wheat grain, and was the most significantly connected with that trait [56]. Combining results of the two studies, we should conclude that the presence of awns could be associated with higher calcium content of wheat grains. This hypothesis has not been tested directly, however if it is true, that association could be useful for wheat biofortification, that is for development mineral-rich crop varieties beneficial for human diet.

#### Plant Heigh

Plant height was expectedly significantly associated with allele variants of reduced-height genes, *Rht-D1b* (*Rht2*), and *Rht-B1e* (*Rht11*). We added these polymorphisms detected using PCR markers as IAB07969 and IAB60001 to the SNP-seq data used for GWAS. Interestingly, that *Rht-B1b* allele marker that was introduced to the set as IAB07477 was not significantly associated with plant height in the winter wheat collection despite the presence of marker variation. In addition to these well-studied polymorphisms, two more SNP-seq polymorphisms were significantly associated with plant height, both located on chromosome 7B – IAB57498 (chr7B: 407,491,560) and IAB28956 (chr7B: 555,163,428). To date, two reduced-height genes were located on 7B chromosome of wheat – *Rht13* at about 714 Mbp of chromosome 7B (absent from Chinese Spring genome, but is most similar to its TraesCS7B02G452600 gene, and is located approximately the same region of chromosome in CDC Stanley wheat genome) [68] and *TaDHL-7B* (TraesCS7B02G055300) located at about 58.7 Mbp on 7B [69]. Both these reduced-height genes on 7B are far away from the significant loci found in our study. Both IAB57498 and IAB28956 are located in regions with no confidence genes annotated nearby in RefSeq1.1 genome of Chinese Spring. Thus, no candidate genes could be readily suggested for these marker-trait associations.

#### Heading and flowering

Period from sowing to heading was expectedly significantly associated with *Photoperiod-D1* (*PPD-D1*) PCR marker, detecting *Ppd-D1a* and *Ppd-D1b* alleles [37], that was added to SNP-seq marker data as IAB04062. *PPD-D1* on chromosome 2D is a major photoperiod response locus in wheat, a mutation of which provides early flowering of plants irrespective to day length and adaptability to broad range of environments [70]. In our GWAS, *PPD-D1* was also significantly associated with 1000 kernel weight and, at a border of significance, with grain yield per unit area (tons per hectare). This result is in agreement with previous studies showing benefits of photoperiod neutrality of wheat plants under short-day conditions, that are observed during growing season in southern regions of Russia [21]. Additionally, a period from sowing to heading in our GWAS was significantly associated with a polymorphism IAB00955 (chr1B: 106,765,881), located in TraesCS1B02G099100 gene coding a diacylglycerol O-acyltransferase enzyme, which itself is unlikely a good candidate. Among the known genes regulating flowering time, *TaELF3-B1 (EARLY FLOWERING 3)* is located on chromosome 1B (TraesCS1B02G477400). However, this one gene is located far away from the IAB00955 marker.

#### Grain appearance score

A polymorphism IAB19354 (chr2D: 516,926,694), located within the 3’-UTR of the TraesCS2D02G402100 gene, coding a polygalacturonase enzyme, was significantly associated with a grain visual score. The grain visual score reflects the likelihood that a breeding line will be selected by a breeder based on an arbitrary estimation of its grain characteristics. Mainly, this score is positively correlated with grain plumpness and negatively to grain shriveling. The presence of disease, pre-harvest sprouting, or other damage can lower this score. According to Wheat Expression Browser (www.wheat-expression.com) TraesCS2D02G402100 gene of polygalacturonase is expressed mainly in grain, and its expression is enhanced under condition of disease [71]. Thus, this gene itself can be a good candidate gene for grain visual score trait. Polygalacturonase is a major hydrolase involved in the degradation of pectin in cell separation events including pod and anther dehiscence, pollen grain maturation, and pollen tube growth [72]. Wheat affected by Fusarium Head Blight (FHB) produces shriveled kernels. Genetic engineering introduction of a polygalacturonase inhibiting protein from green beans into all the tissues of wheat plants reduces FHB damage in wheat grains [73]. Thus, we can speculate that a polygalacturonase gene TraesCS2D02G402100 could be involved in grain damage during some physiological disorder like pre-harvest sprouting, or in pathogenic process being induced by presence of fungi, and a mutation of this gene could result in damage tolerance, providing more healthy-looking grain. Interestingly, a QTL for resistance to FHB was observed on chromosome 2D not far from the locus of IAB19354 marker [74].

IAB32845 on chromosome 1B (chr1B: 676,812,396), is another marker that was associated with grain visual score in our study. Some previous studies have detected a QTL for grain filling in the same region of chromosome 1B [75]. The polymorphism IAB32845 is located within the TraesCS1B02G466400 gene. This gene, according to International Wheat Information System (www.wheatis.org), encodes a putative leaf rust disease resistance protein [76], however according to Wheat Expression Browser it is expressed in roots. A marker IAB32845 itself tags a missense mutation (p.Asp733Gly), however SIFT (Sorting Intolerant From Tolerant) [77] predicts this amino-acid base change to be tolerated with a score of 0.42. In this case the direct connection of a gene with the trait should not be assumed, and the association found may be explained by linkage with some other gene.

#### Grain test weight

An SNP on chromosome 3B (IAB41988, chr3B: 655,860,618) was near-significantly associated with grain test weight. Test weigh is a mass of a known volume of grain in bulk. It is a valuable quality indicator connected with grain moisture content, flour extraction rate and bread-making quality. This is a complex trait, determined by grain weight, shape and smoothness of its surfaces. Earlier 4 QTL were found for test weight on chromosome 3B [78], one of which (PRT_3B.2, chr3B: 564,248,743) is located at least in the same 10-th part of chromosome as IAB41988 marker. A candidate gene *TaMYB10-B1* was suggested for PRT_3B.2, that is associated with grain color and pre-harvest sprouting tolerance [78]. However red grain color gene *TaMYB10-B1* (TraesCS3B02G515900, chr3B: 757,918,264) is located far from both IAB41988 marker and PRT_3B.2 QTL. An SNP itself is characterized as a missense variant in TraesCS3B01G419400 gene encoding EKC/KEOPS complex subunit bud32 involved in tRNA universal modification, however this one gene missing from the newer annotation version 1.1. Thus, we cannot suggest a good candidate gene for this marker-trait association.

#### Grain starch content

Almost significant marker-trait association was observed for starch content of the grain and a marker IAB08754 (chr5A: 520,117,260) in TraesCS5A02G307500 gene coding a peroxisomal acyl-activating enzyme 5. Previously a conditional QTL for content of a starch component amylopectin, that was observed only under irrigation, was detected on chromosome 5 A (QAmp-5 A.3) almost in the same region as IAB08754 in our study [79]. A starch-biosynthesis-regulating gene TraesCS5A01G308400 coding a transcription factor similar to maize *ZmbZIP91* and rice *OsbZIP30* (*RF2b*) and located at about 900Kb distance from IAB08754 is a probable candidate for this locus [80]. According to Wheat Expression Browser TraesCS5A01G308400 gene is mainly expressed in grain and other spike tissues.

#### Leaf rust damage

A polymorphism IAB17595 (chr2A: 717,188,839) causing missense mutation (p.Trp213Gly) in TraesCS2A02G478600 gene encoding a MONOSACCHARIDE TRANSPORTER 1 protein was significantly associated with quantitative estimation of leaf rust damage (caused by biotrophic fungus *Puccinia recondita* f. sp. *tritici*) to wheat plants expressed as a percentage of leaf area occulated by the disease. Many leaf rust (*Lr*) resistance genes are known to be located on the short arm chromosome 2 A in wheat, including *Lr17a*,* Lr37*,* Lr45*,* Lr65*,* Lr81 and LrM.* Some of these genes are parts of alien genetic material passed into wheat genome in the forms of translocations, like *Lr37* which is a part of 2NS/2AS translocation from *Aegilops ventricosa*,* LrM* gene from *Aegilops markgrafii*, and *Lr45* from *Secale cereale* (rye) [81]. Some accessions of our collection may possibly possess translocations involving 2AS chromosome arm, which could be suggested based on missing information from a group of terminal markers in some accessions. However, IAB17595 is located in other chromosome part, most probably in long chromosome arm. Several previous studies identified QTLs for rust disease resistance on long arm of chromosome 2 A in wheat [81]. IAB17595 polymorphism is located between two of those QTLs: QLr.ifa-2AL at *Xgwm312* microsatellite marker [82], and QLr.hebau-2AL at *wmc181* marker [83]. QTL QLr.ifa-2AL provides resistance to both leaf rust and stripe rust simultaneously [82]. However, no candidate genes were suggested for those loci. Interestingly, that sugar transporter gene TraesCS2A02G478600, a mutation in which was associated with rust resistance in our study, is expressed in leaves and significantly changes its expression in response to stripe rust inoculation (www.wheat-expression.com). Based on this, and some previous studies showing the role of sugar transporters in fungal disease resistance [84], we can hypothesize that sugar transporter gene TraesCS2A02G478600 can itself be a candidate gene for quantitative leaf rust resistance, regulating nutrition of parasitic fungus. Other candidates from this region could be TraesCS2A02G477700 gene, which is expressed at high level and codes the NON-SPECIFIC LIPID-TRANSFER PROTEIN, and TraesCS2A02G481400 gene coding (2 S)-flavan-3-ol-forming ANTHOCYANIDIN REDUCTASE. The non-specific lipid-transfer proteins are known for their antimicrobial activity due to ability to disrupt the permeability of the pathogens’ outer membranes [85]. While flavan-3-ols are also effective antimicrobial substances acting against rust pathogens [86]. Further fine-mapping studies will be needed to find a real causal gene for this rust resistance locus.

#### Septoria blotch damage

The flag leaf damage by *Septoria tritici* blotch caused by the fungus *Zymoseptoria tritici* was associated with IAB13554 polymorphism (chr7B: 134,556,005) in 2019. Several QTL on the long arm of chromosome 7B, including *Stb8* gene were identified in bread and durum wheat [87, 88]. However, the IAB13554 polymorphism lies far from them, possibly in different (short) chromosome arm. The synonymous polymorphism IAB13554 associated with *Septoria tritici* blotch is located within TraesCS7B02G115900 gene coding an aspartic protease. Aspartic proteases are proteolytic enzymes with an aspartate residue (Asp) in their active site, involved a broad spectrum of biological roles in plants, including defense responses against a diversity of stresses, including interaction with pathogens [89, 90]. TraesCS7B02G115900 is expressed in most tissues except grain, and is activated by treatment with pathogen-associated molecular patterns, like chitin or flagellin (www.wheat-expression.com). Thus, TraesCS7B02G115900 gene may be taken itself as a candidate for *Septoria tritici* blotch resistance.

### General GWAS results

Some marker-trait associations that were discovered in our study were previously known. This provided validation for our genotyping and association finding procedure. Due to advances in crop genetics and the increased use of genome-wide association studies (GWAS), there are often a large number of genes and markers associated with a particular trait, and selecting markers for breeding can be a challenging task. Even if a GWAS identifies a previously known association, the presence of that association in a specific breeding population is still beneficial information.

A major part of our final SNP set were mutations in protein-coding genes that cause changes to amino-acid sequence or disrupt protein function. Our study shows that the concept of targeting protein-changing mutations was equal or less successful, than targeting some random SNPs for finding marker-trait associations. It seems, that this would be true not only for wheat, but to a broad range of species, including humans [91]. Among SNPs associated with various biological traits in humans via GWAS, the vast majority of mutations, about 94%, are located within the noncoding genomic regions [92]. This may reflect the significant role of the non-coding part of the genome in shaping the phenotype of living organisms by determining the patterns of gene expression. This means that the blueprint of the organism, such as where and when certain genes should be expressed, is more significant than the individual components that make it up, such as proteins. In other words, different shapes can be created from the same basic building blocks. The SNPs that showed significant associations with traits in previous GWAS studies were also not more successful in finding associations in a new study than random SNPs. This could be explained by the complex genetic control of traits, different genetic variations in collections, and the effect of genotype-environment interactions.

Some traits in our study, like grain protein content or lodging resistance, did not find significant marker-trait associations, despite genetic variation of them in a collection is definitely present. This is likely a result of the low density of the markers on chromosomes in a final SNP set. Other reason could be the chosen GWAS method. Mixed linear models (MLM) method, that we used in our study, controls the population structure and unequal relatedness among individuals within subpopulations. However, this method may yield false negative results due to overcorrection or when causal variants are in linkage disequilibrium [93]. There are newer methods with higher statistical power, that are able to solve this problem, like the multiple loci mixed model (MLMM), fixed and random model circulating probability unification (FarmCPU) or Bayesian information and linkage disequilibrium iteratively nested keyway (BLINK), and the last model is considered the best to date [94, 95].

The panel 3.5 thousand of high-quality SNP-seq markers that was developed in our study is unlikely itself a good choice for a new GWAS, but could be used in combination with markers from other panels. Also, it would be valuable for rough-mapping QTL (quantitative trait locus) studies based on crosses, as they generally require less dense genetic maps. Separate SNP-seq markers that showed significant marker-trait associations in our study were successfully converted by us into KASP markers. These KASP markers confirmed their allele-discrimination ability and can be readily used by breeders for marker assisted selection.

## Conclusion

This study applied a 16,500-SNP panel to characterize 200 winter wheat accessions from Krasnodar, revealing clear population structure and untapped opportunities to enrich the national gene pool with favorable alleles for yield, grain quality, and stress resistance. SNP-seq proved effective for detecting structural variants, validating key loci such as *Rht*, *Ppd*, and *Vrn-D1*, and uncovering novel associations, including a potential link between awns and grain calcium content. Several unique trait-linked markers absent from existing arrays were identified, underscoring the added value of this resource. These findings provide a strong foundation for functional validation and the development of breeder-friendly KASP markers to accelerate genetic improvement in wheat.

## Supplementary Information

Supplementary Material 1. Additional file 1.pdf supplementary figures and tables. Supplementary figure 1. Proportion of missing (no-call) values for each of the 16.5 K SNPs mapped on chromosomes pseudomolecules of RefSeq1.0 wheat genome assembly; Supplementary figure 2. Plots showing the distribution of SNPs among chromosomes and the gradual decrease their number on various stages of filtering; Supplementary figure 3. Geographical origin of winter wheat accessions on the scatterplot of principal components (PC1 – PC2); Supplementary figure 4. The geographical origin of wheat accessions shown on the neighbor-joining tree; Supplementary figure 5. Manhattan-plots and QQ-plots resulting from GWAS; Supplementary figure 6. Boxplots for significant marker-trait associations found in GWAS; Supplementary figure 7. KASP markers visualization; Supplementary table 1. A list of studies that were automatically screened for mentioning polymorphisms from wheat iSelect 90k array; Supplementary table 2. The KASP primers for detecting SNP alleles; Supplementary table 3. Comparison the KASP markers results and SNP-Seq. Additional file 2.xlsx Initial 60 K SNP set: positions and short descriptions of 60 thousand initial SNPs selected for SNP-seq marker panel design. Additional file 3.xlsx Oligonucleotide probes used for target DNA fragments enrichment. Additional file 4.xlsx Correspondence between sequencing samples and wheat accessions. Additional file 5.xlsx Phenotype BLUES used for GWAS. Additional file 6.xlsx Genotypes of 200 winter wheat accessions (16.497 SNP-seq markers). Additional file 7.xlsx 1RS.1BL translocation prediction and validation. Additional file 8.xlsx Diversity of specific sequencing-genotyped SNPs that are commonly used as breeding markers. Additional file 9.xlsx Significant marker-trait associations.

## Data Availability

The raw sequencing data generated and analyzed during the current study are available in the NCBI SRA repository, https://www.ncbi.nlm.nih.gov/sra/PRJNA1270396. Other data generated or analyzed during this study are included in this published article and its supplementary information files.
