# Supplementary material for: Genome-wide association study of agronomic traits in winter wheat (Triticum aestivum L.) using a custom SNP marker set
Source: BMC Plant Biol. 2025 Oct 1;25:1279. doi: 10.1186/s12870-025-07322-y (PMC12487310; doi:10.1186/s12870-025-07322-y)
Supplement: Supplementary file 1 — Supplementary Material 1. Additional file 1.pdf supplementary figures and tables. Supplementary figure 1. Proportion of missing (no-call) values for each of the 16.5 K SNPs mapped on chromosomes pseudomolecules of RefSeq1.0 wheat genome assembly; Supplementary figure 2. Plots showing the distribution of SNPs among chromosomes and the gradual decrease their number on various stages of filtering; Supplementary figure 3. Geographical origin of winter wheat accessions on the scatterplot of principal components (PC1 – PC2); Supplementary figure 4. The geographical origin of wheat accessions shown on the neighbor-joining tree; Supplementary figure 5. Manhattan-plots and QQ-plots resulting from GWAS; Supplementary figure 6. Boxplots for significant marker-trait associations found in GWAS; Supplementary figure 7. KASP markers visualization; Supplementary table 1. A list of studies that were automatically screened for mentioning polymorphisms from wheat iSelect 90k array; Supplementary table 2. The KASP primers for detecting SNP alleles; Supplementary table 3. Comparison the KASP markers results and SNP-Seq. Additional file 2.xlsx Initial 60 K SNP set: positions and short descriptions of 60 thousand initial SNPs selected for SNP-seq marker panel design. Additional file 3.xlsx Oligonucleotide probes used for target DNA fragments enrichment. Additional file 4.xlsx Correspondence between sequencing samples and wheat accessions. Additional file 5.xlsx Phenotype BLUES used for GWAS. Additional file 6.xlsx Genotypes of 200 winter wheat accessions (16.497 SNP-seq markers). Additional file 7.xlsx 1RS.1BL translocation prediction and validation. Additional file 8.xlsx Diversity of specific sequencing-genotyped SNPs that are commonly used as breeding markers. Additional file 9.xlsx Significant marker-trait associations. [file 12870_2025_7322_MOESM1_ESM.zip › Additional file 1.pdf]

Supplementary figures and tables

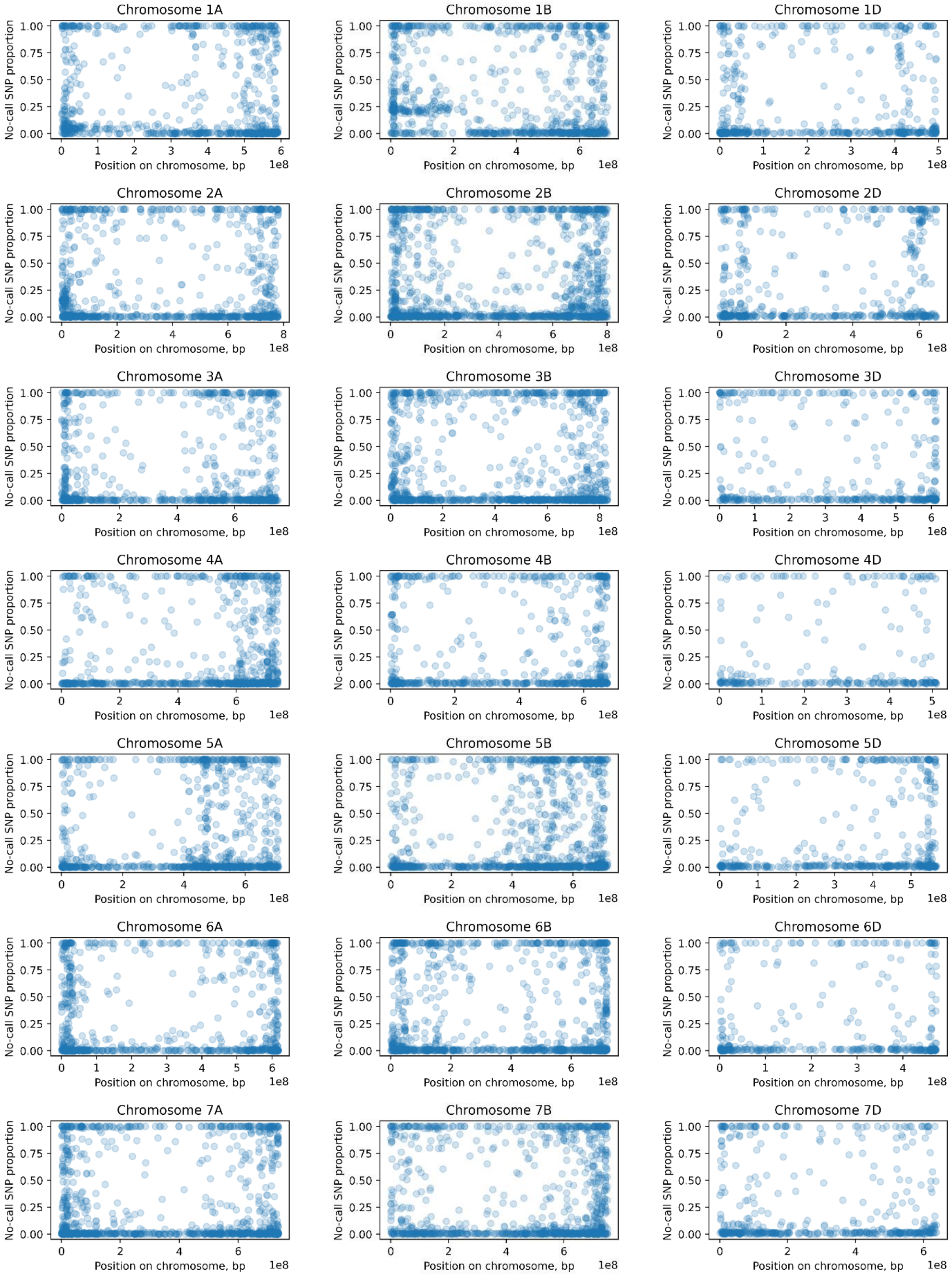

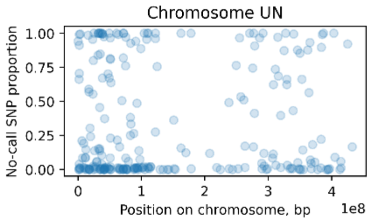

Supplementary figure 1. Proportion of missing (no-call) values for each of the 16.5K SNPs mapped on chromosomes pseudomolecules of RefSeq1.0 wheat genome assembly. Chromosome UN is an assembly of unmapped contigs.

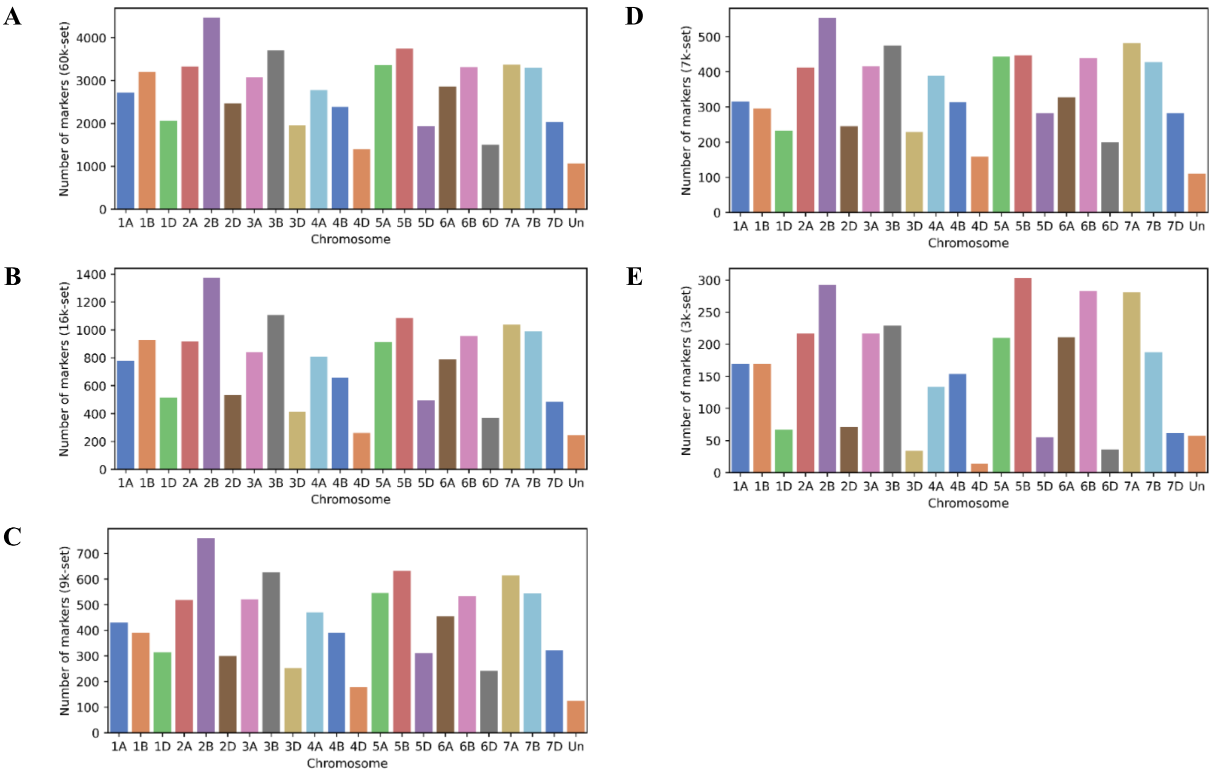

Supplementary figure 2. Plots showing the distribution of SNPs among chromosomes and the gradual decrease their number on various stages of filtering. Initially 60 thousand SNPs were selected (A), of them only 16.5 thousand were suitable for specific primers development (B), of them only 9.5 thousand were successfully called in more than 90% of wheat accessions (C), of those 7.5 thousand were enough locus-specific (D), and finally only 3.5 thousand showed minor allele frequency more than 5% (E).

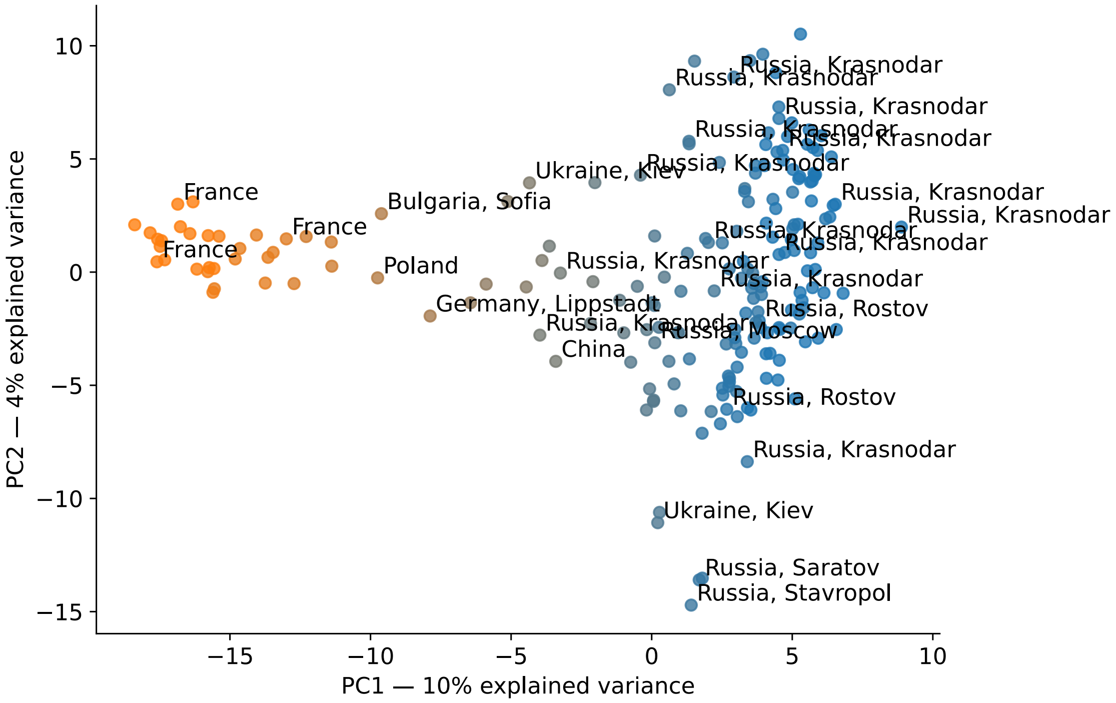

Supplementary figure 3. Geographical origin of winter wheat accessions on the scatterplot of principal components (PC1 – PC2).

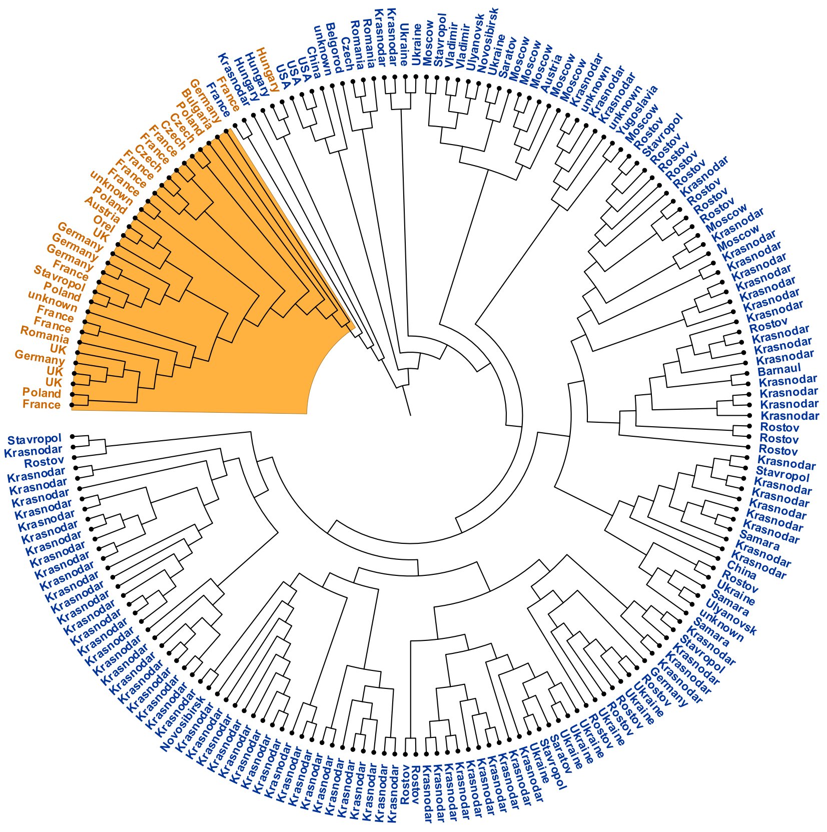

Supplementary figure 4. The geographical origin of wheat accessions shown on the neighbor-joining tree. Only the city is shown for accessions from Russia, and only the country is shown for accessions from other countries

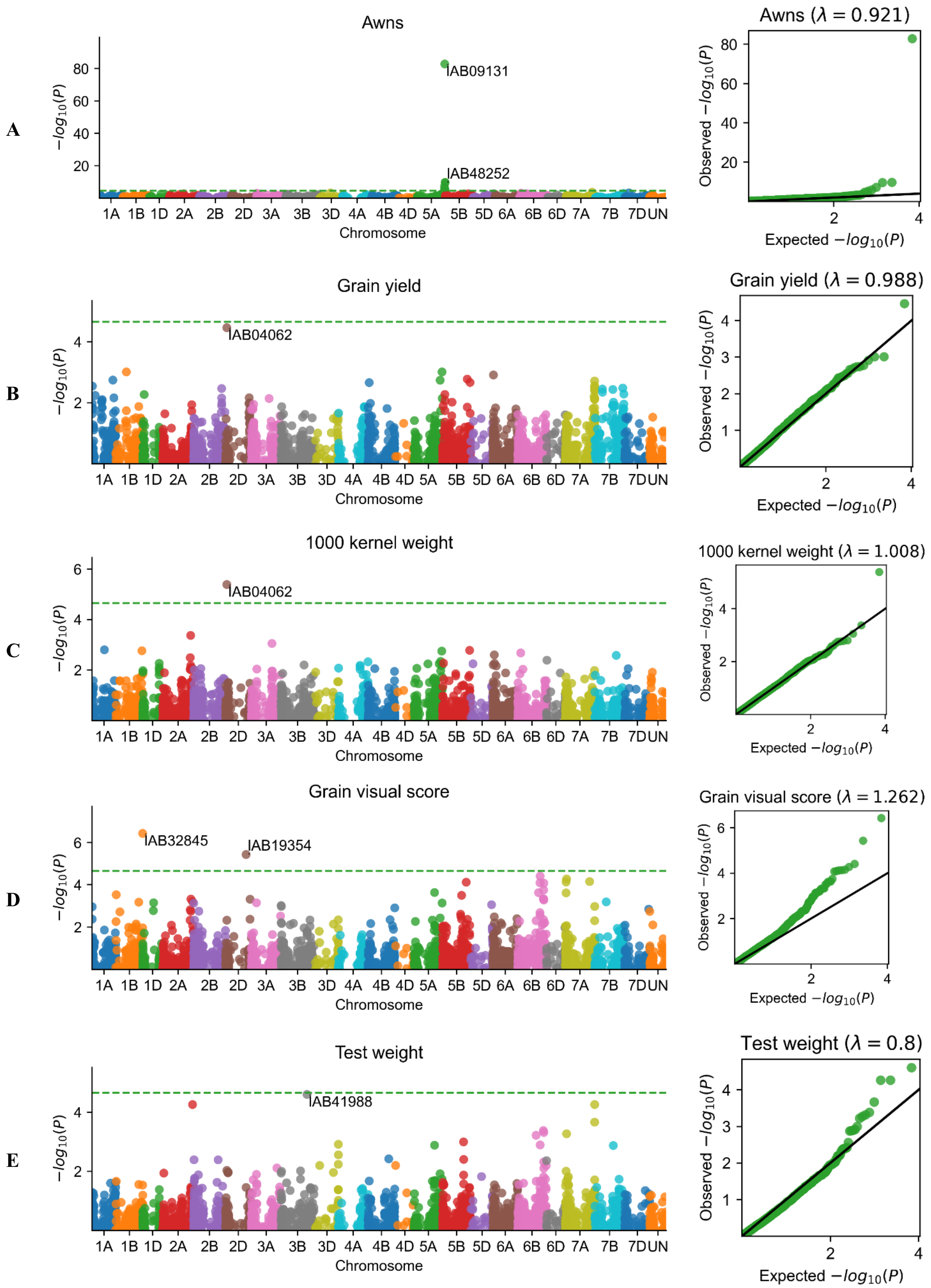
A
Awns
IAB09131
IAB48252
Awns (λ = 0.921)
B
Grain yield
IAB04062
Grain yield (λ = 0.988)
C
1000 kernel weight
IAB04062
1000 kernel weight (λ = 1.008)
D
Grain visual score
IAB32845
IAB19354
Grain visual score (λ = 1.262)
E
Test weight
IAB41988
Test weight (λ = 0.8)
$-log_{10}(P)$
Chromosome
1A 1B 1D 2A 2B 2D 3A 3B 3D 4A 4B 4D 5A 5B 5D 6A 6B 6D 7A 7B 7DUN
Observed $-log_{10}(P)$
Expected $-log_{10}(P)$

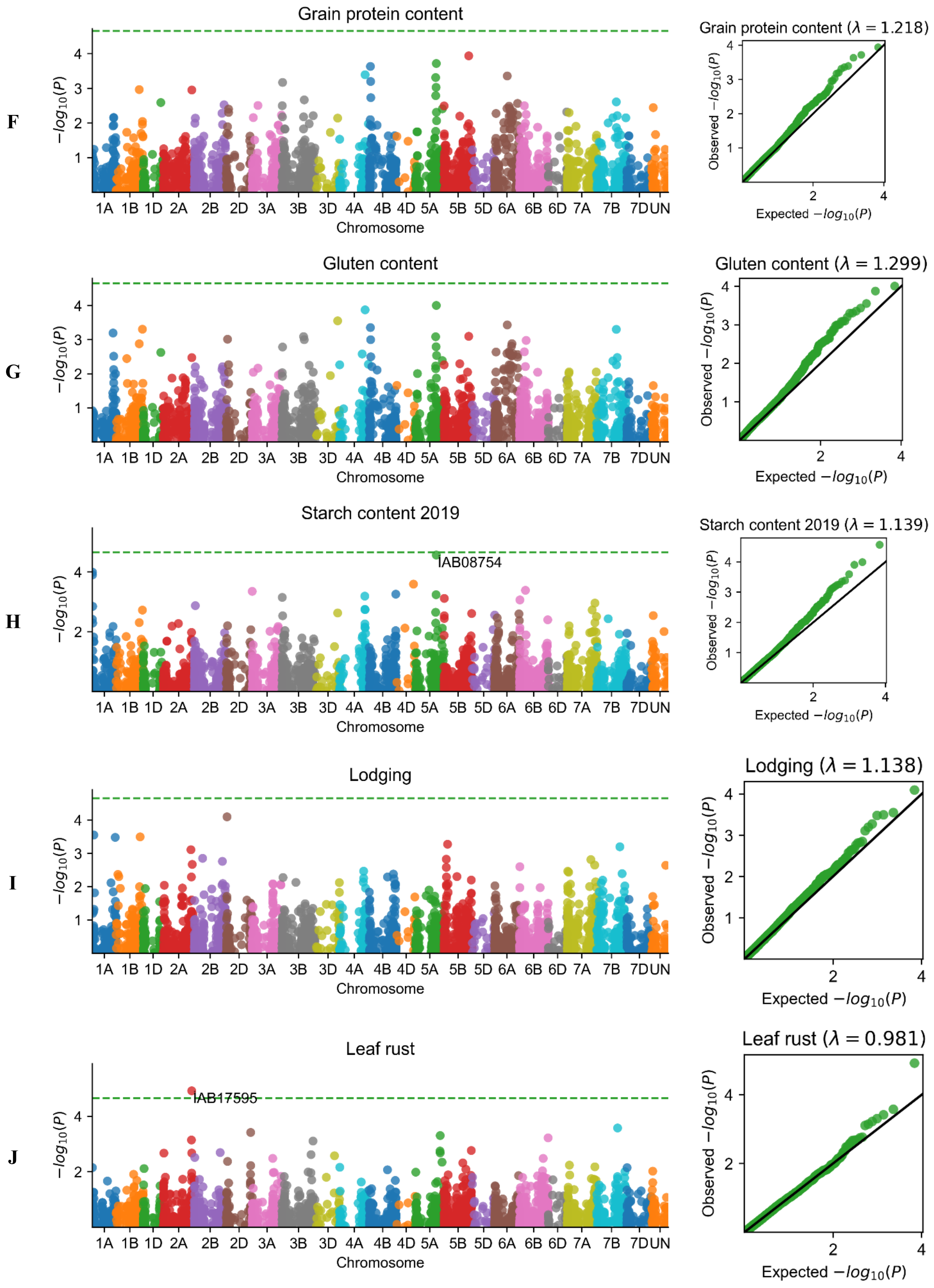
F
G
H
I
J
Grain protein content
Grain protein content (λ = 1.218)
Gluten content
Gluten content (λ = 1.299)
Starch content 2019
Starch content 2019 (λ = 1.139)
IAB08754
Lodging
Lodging (λ = 1.138)
Leaf rust
Leaf rust (λ = 0.981)
IAB17595
$-log_{10}(P)$
Observed $-log_{10}(P)$
Expected $-log_{10}(P)$
1A 1B 1D 2A 2B 2D 3A 3B 3D 4A 4B 4D 5A 5B 5D 6A 6B 6D 7A 7B 7D UN
Chromosome

K

L

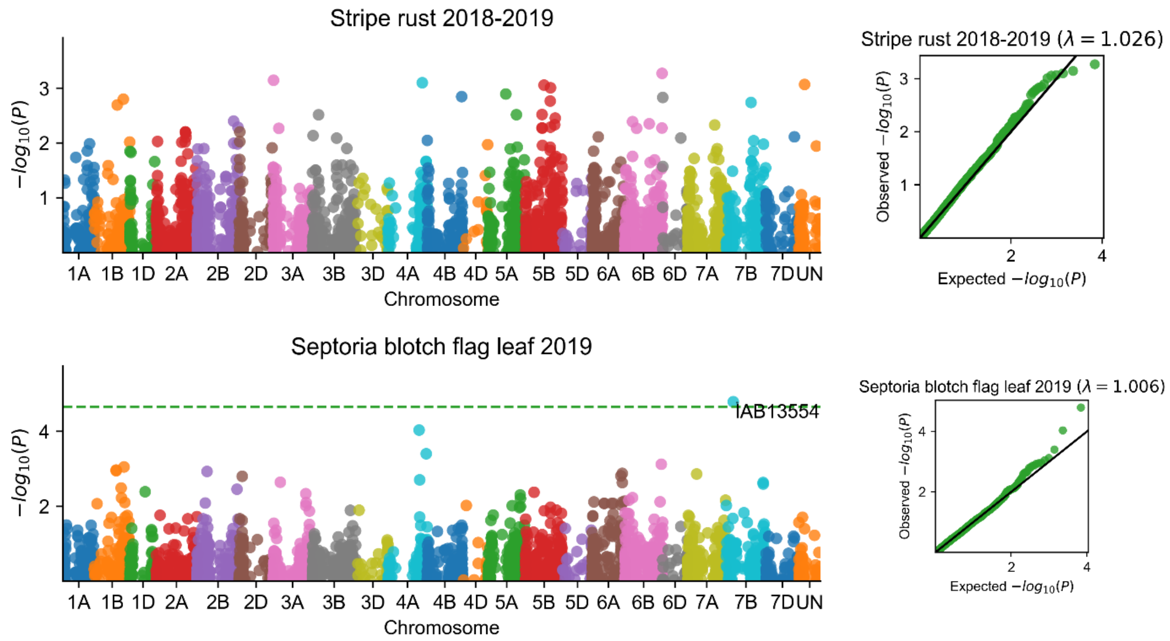

(1) **(2)**

Supplementary figure 5. Manhattan-plots (1) and QQ-plots (2) resulting from GWAS. Manhattan-plots show marker-trait associations. The green dashed line on Manhattan-plots plots divides significant associations (above) from non-significant associations (below) based on Bonferroni correction for initial α=0.05 and effective number of markers equal to 2286 calculated using SimpleM software. The QQ-plots (2) show deviation of significance of associations from the expected by random coincidence (points high above the trend line indicate the presence of non-random associations).

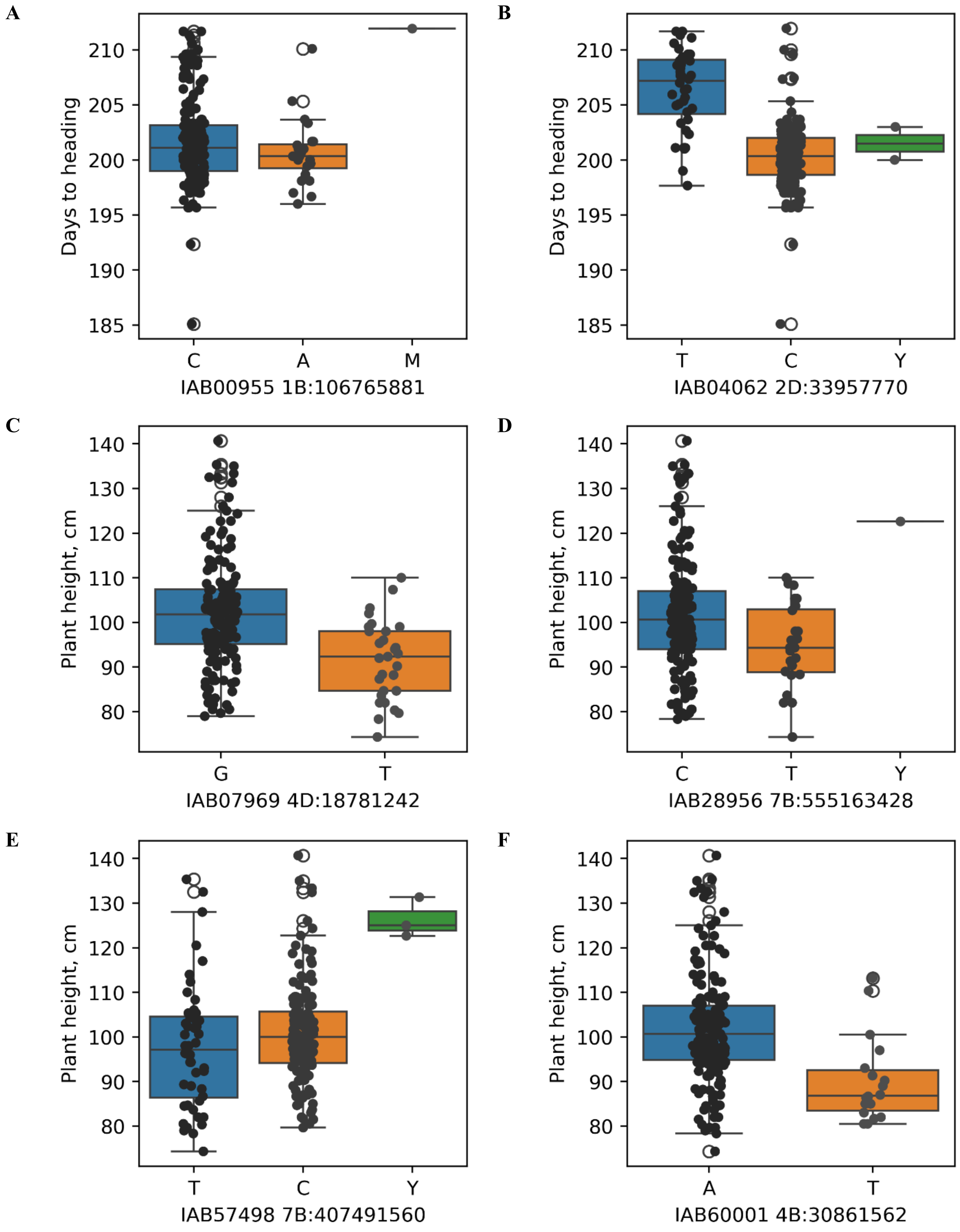
A
B
Days to heading
210
205
200
195
190
185
C
A
M
IAB00955 1B:106765881
T
C
Y
IAB04062 2D:33957770
C
D
Plant height, cm
140
130
120
110
100
90
80
G
T
IAB07969 4D:18781242
C
T
Y
IAB28956 7B:555163428
E
F
T
C
Y
IAB57498 7B:407491560
A
T
IAB60001 4B:30861562

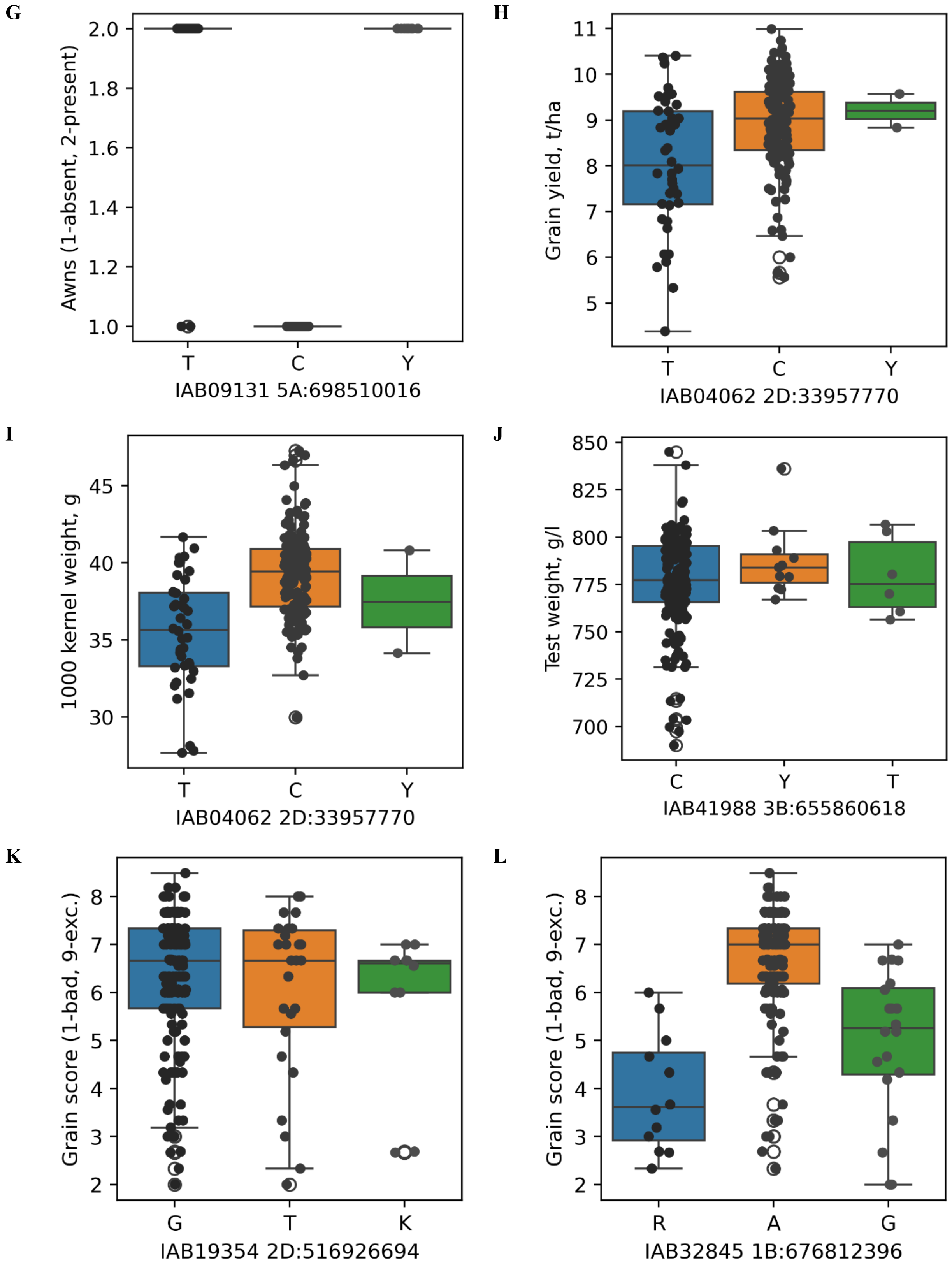
G
Awns (1-absent, 2-present)
IAB09131 5A:698510016
H
Grain yield, t/ha
IAB04062 2D:33957770
I
1000 kernel weight, g
IAB04062 2D:33957770
J
Test weight, g/l
IAB41988 3B:655860618
K
Grain score (1-bad, 9-exc.)
IAB19354 2D:516926694
L
Grain score (1-bad, 9-exc.)
IAB32845 1B:676812396

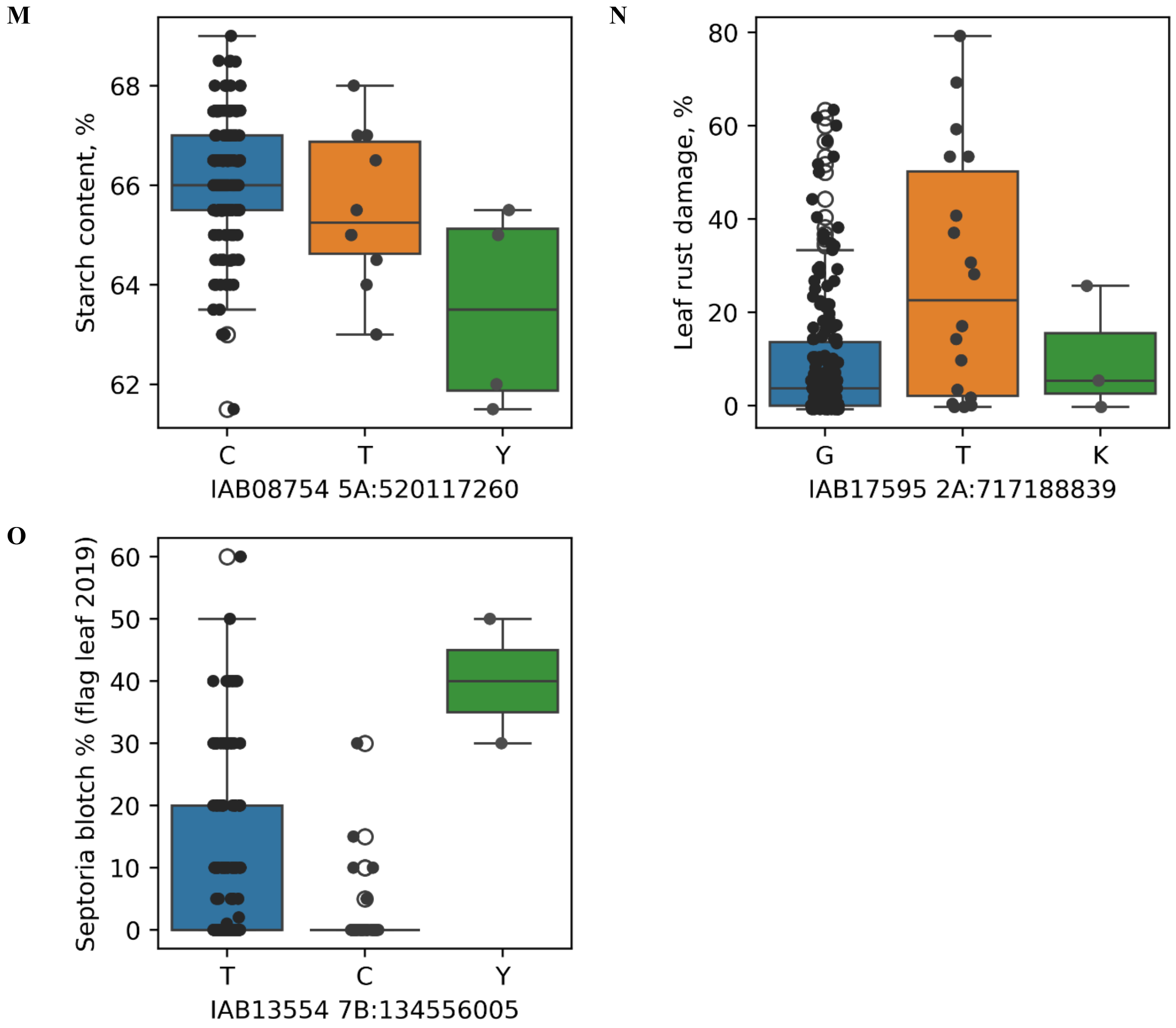

Supplementary figure 6. Boxplots for significant marker-trait associations found in GWAS. Plots showing phenotypes (three-year BLUEs) depending on SNP genotypes in a winter wheat collection of 200 accessions. On the plots, boxes extend from the first to the third quartile of the data, with lines within the boxes showing medians. Whiskers extend from each box to the farthest data point within 1.5 times the interquartile range, with outlier points shown as hollow circles. Dark points represent individual wheat accession values, while SNP genotypes are indicated along the horizontal axis. The name and position of the marker in the Chinese Spring wheat RefSeq1.1 genome is indicated on axes labels. Vertical axes represent traits: days from sowing to heading (a, b), plant height (c, d, e, f), the presence of awns (g), grain yield (h), 1,000-kernel weight (i), test weight (j), visual score for grains (k, l), starch content in grains (m), damage from leaf rust (n), and Septoria blotch on flag leaves in 2019 (o).

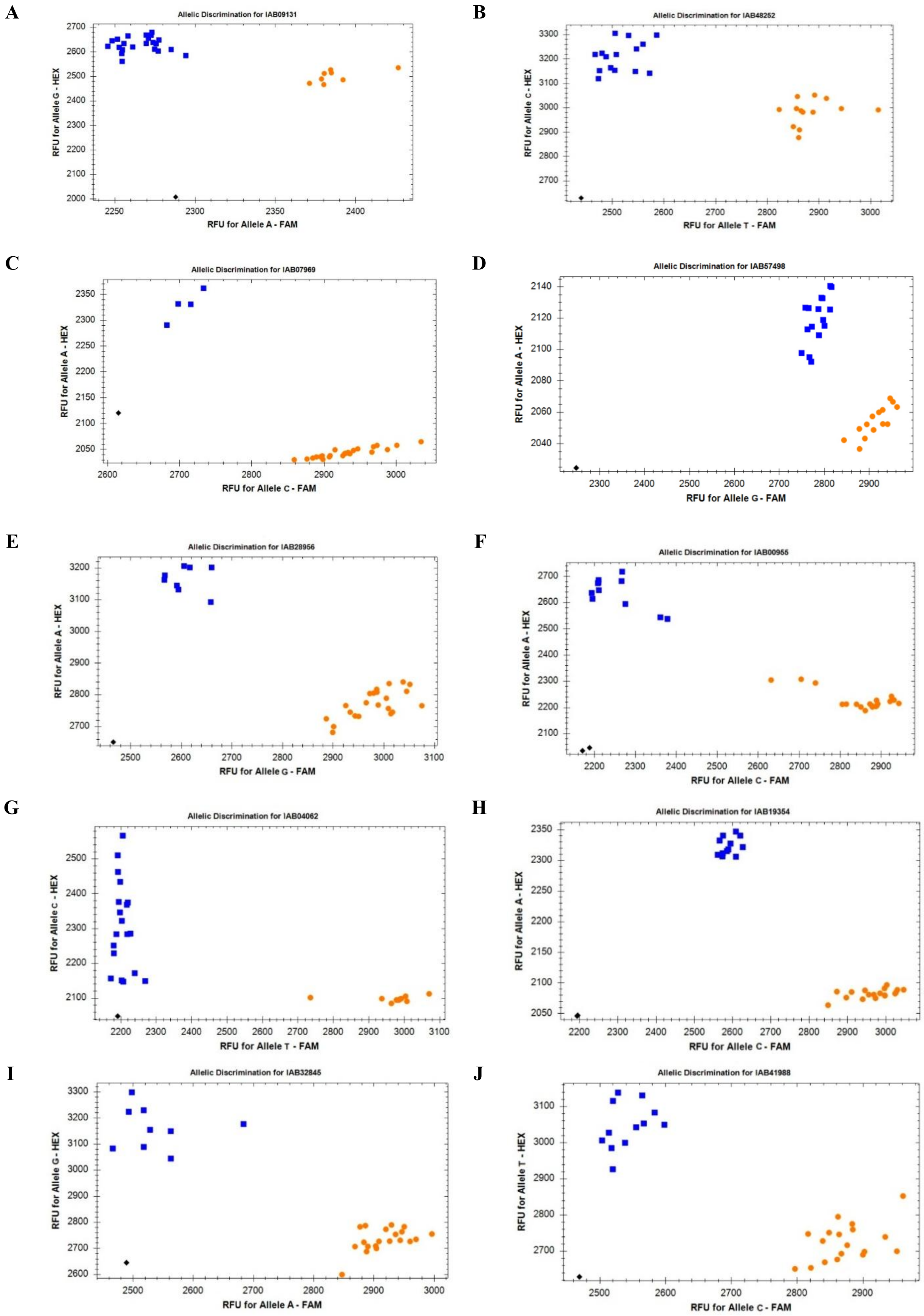
A
Allelic Discrimination for IAB09131
RFU for Allele G - HEX
RFU for Allele A - FAM
B
Allelic Discrimination for IAB48252
RFU for Allele C - HEX
RFU for Allele T - FAM
C
Allelic Discrimination for IAB07969
RFU for Allele A - HEX
RFU for Allele C - FAM
D
Allelic Discrimination for IAB57498
RFU for Allele A - HEX
RFU for Allele G - FAM
E
Allelic Discrimination for IAB28956
RFU for Allele A - HEX
RFU for Allele G - FAM
F
Allelic Discrimination for IAB00955
RFU for Allele A - HEX
RFU for Allele C - FAM
G
Allelic Discrimination for IAB04062
RFU for Allele C - HEX
RFU for Allele T - FAM
H
Allelic Discrimination for IAB19354
RFU for Allele A - HEX
RFU for Allele C - FAM
I
Allelic Discrimination for IAB32845
RFU for Allele G - HEX
RFU for Allele A - FAM
J
Allelic Discrimination for IAB41988
RFU for Allele T - HEX
RFU for Allele C - FAM

K

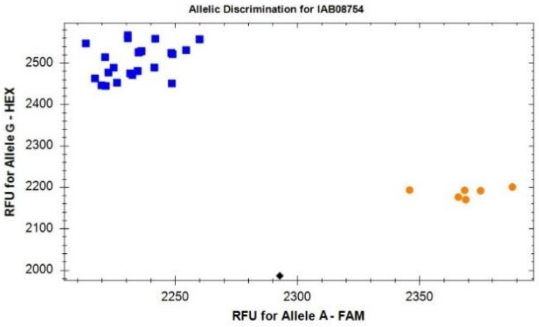

L

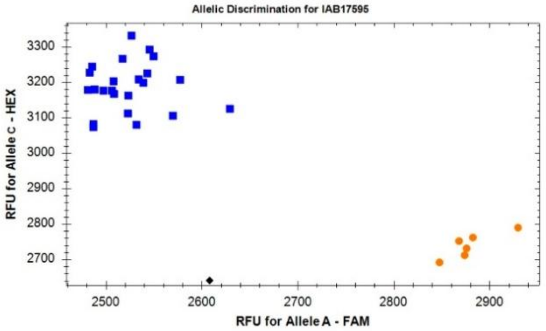

M

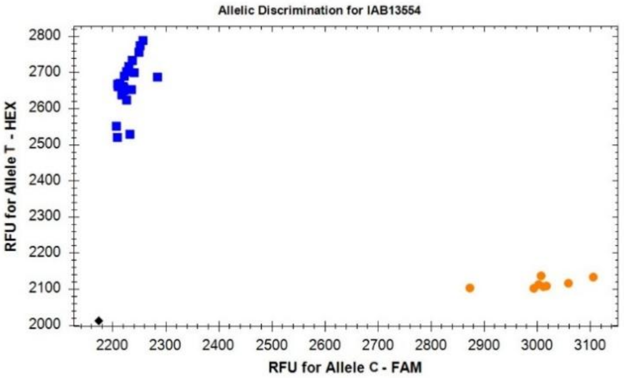

Supplementary figure 7. KASP markers visualization. DNA samples with different SNP alleles are denoted by blue squares and orange circles. A control sample without DNA template is represented by a black diamond. RFU stands for relative fluorescence units.

Supplementary table 1. A list of studies that were automatically screened for mentioning polymorphisms from wheat iSelect 90k array (potentially linked to valuable traits in wheat)

**Traits**	**Reference***
Grain yield and related traits	[1]
Stripe rust resistance	[2]
1000 kernel weight	[3]
Grain yield	[4]
Physiological traits	[5]
Resistance to pre-harvest sprouting and grain color	[6]
Leaf rust resistance	[7]
Soluble carbohydrates in stem	[8]
Domestication-related traits	[9]
Black point reaction	[10]
Grain yield and related traits	[11]
Leaf rust, yellow (stripe) rust, common bunt, and tan spot	[12]
Stripe rust resistance	[13]
Kernel number per spike	[14]
Calcium accumulation in grain	[15]
Yield and quality-related traits	[16]
Grain yield and related traits	[17]
Zinc concentration in grain	[18]
Seed dormancy	[19]
36 agronomical traits	[20]

* The list of references can be found at the end of this supplementary file.

Supplementary table 2. The KASP primers for detecting SNP alleles.

SNP	Primer	Sequence (5'->3') *	SNP	Primer	Sequence (5'->3')
	A	GAGCTCTTTCTGAACCCCCA		A	GCGGGCCTAGATCAGGGC
IAB09131	B	GAGCTCTTTCTGAACCCCCG	IAB19354	B	GCGGGCCTAGATCAGGGA
	C	CCCGACTGTATTTAACAGCAAGC		C	GTCTACCCCAAGATCCCTTGC
	A	CGATCAGGGTCATACACCCT		A	CACTTATCTCTTGTATGGGGTGA
IAB48252	B	CGATCAGGGTCATACACCCC	IAB32845	B	CACTTATCTCTTGTATGGGGTGG
	C	CCCGACTGTATTTAACAGCAAGC		C	GCTGATCCAGTTCGTCAATGC
	A	TGGCCATCTCGAGCTGCTC		A	GAACACCAAGCCGCCTTGC
IAB07969	B	TGGCCATCTCGAGCTGCTA	IAB41988	B	GAACACCAAGCCGCCTTGT
	C	GCGGACGTGGCGCAGAAG		C	CTTACCTCGGTCTCCCTTCC
	A	AATCCATGGCGTGGTCGCG		A	TGCAGGGGTACTTCAACGACA
IAB57498	B	AATCCATGGCGTGGTCGCA	IAB08754	B	TGCAGGGGTACTTCAACGACG
	C	GGGTAGCAAGGTCTCTTGTGC		C	GTAGAACCAGCCGTCTCGC
	A	GGCACCTCATTTACTTCATGCG		A	CATTTTTTGCCACGATGCTCCA
IAB28956	B	GGCACCTCATTTACTTCATGCA	IAB17595	B	CATTTTTTGCCACGATGCTCCC
	C	CCGACATAGTTCTAGCAGACATC		C	GATCAGCTTCAAGGGATGGAAC
	A	CTTGGATGATGTTGGCACCATC		A	GACCTCATCCTTTATACAAGCCC
IAB00955	B	CTTGGATGATGTTGGCACCATA	IAB13554	B	GACCTCATCCTTTATACAAGCCT
	C	CGTCACCATCTCCGATGAAG		C	CCTGGGGAGAACCATGTTTTC
	A	CAAGGAAGTATGAGCAGCGGTT			
IAB04062	B	CAAGGAAGTATGAGCAGCGGTC			
	C	GACGCCTCCCACTACACTG			

*Here only SNP-specific parts of primers A and B are shown. The 5'-ends of primers A should be extended by the sequence 5'-gaaggtcggagtcaacggatt-3', while 5'-ends of primers B by the sequence 5'-gaaggtgaccaagttcatgct-3', which are needed for KASP. The SNP-specific nucleotide is underscored.

Supplementary table 3. Comparison the KASP markers results and SNP-Seq.

Marker	IAB09131		IAB48252		IAB07969		IAB57498		IAB28956		IAB00955		IAB04062		IAB19354		IAB32845		IAB41988		IAB08754		IAB17595		IAB13554	
Accession	Seq	KASP	Seq	KASP	Seq	KASP	Seq	KASP	Seq	KASP	Seq	KASP	Seq	KASP	Seq	KASP	Seq	KASP	Seq	KASP	Seq	KASP	Seq	KASP	Seq	KASP
Altigo	A	A	T	T	A	A	A	A	A	A	C	C	T	T	A	A	A	A	C	C	G	G	A	A	C	C
Anastasiia	G	G	C	C	C	C	G	G	G	G	C	C	T	T	C	C	A	A	T	T	G	G	A	A	T	T
Apash	G	G	C	C	C	C	A	A	A	A	C	C	C	C	A	A	A	A	C	C	A	A	C	C	C	C
Aphina	G	G	T	T	C	C	A	A	G	G	A	A	C	C	C	C	A	A	C	C	G	G	C	C	T	T
ACPG 154	A	A	T	T	C	C	A	A	G	G	C	C	T	T	C	C	G	G	C	C	G	G	C	C	T	T
Bezostaya 100	G	G	T	T	C	C	G	G	G	G	C	A*	C	C	A	A	A	A	T	T	G	G	C	C	T	T
Bohemiia	G	G	C	C	C	C	A	A	G	G	C	C	C	C	A	A	G	G	C	C	G	G	C	C	T	T
Deya	G	G	T	T	C	C	G	G	G	G	C	C	C	C	C	C	A	A	T	T	G	G	C	C	T	T
Caphorn	G	G	C	C	A	A	A	A	G	G	C	C	C	C	C	C	G	G	C	C	A	A	C	C	C	C
Krasa Dona	G	G	T	T	C	C	G	G	G	G	A	A	C	C	A	A	A	A	T	T	G	G	C	C	T	T
Krasnoobskaya ozimaya	G	G	C	C	C	C	A	A	G	G	C	C	T	T	C	C	G	G	T	T	G	G	A	A	T	T
Moskovskaya 40	A	A	T	T	C	C	G	G	G	G	C	C	T	T	C	C	A	A	T	T	G	G	C	C	T	T
Sineva	G	G	C	C	C	C	G	G	G	G	C	C	C	C	C	C	G	G	C	C	G	G	C	C	T	T
Favoritka	G	G	C	C	A	A	A	A	A	A	A	A	C	C	C	C	A	A	C	C	G	G	C	C	C	C
Flamenco	A	A	C	C	C	C	G	G	A	A	C	C	C	C	A	A	A	A	C	C	A	A	A	A	T	T

* Asterisk marks the only mismatched result of genotyping.

References

1. Sukumaran S, Dreisigacker S, Lopes M, Chavez P, Reynolds MP. Genome-wide association study for grain yield and related traits in an elite spring wheat population grown in temperate irrigated environments. Theor Appl Genet. 2015;128:353–63.

2. Maccaferri M, Zhang J, Bulli P, Abate Z, Chao S, Cantu D, et al. A Genome-Wide Association Study of Resistance to Stripe Rust (*Puccinia striiformis* f. sp. *tritici*) in a Worldwide Collection of Hexaploid Spring Wheat (*Triticum aestivum* L.). G3amp58 GenesGenomesGenetics. 2015;5:449–65.

3. Zanke CD, Ling J, Plieske J, Kollers S, Ebmeyer E, Korzun V, et al. Analysis of main effect QTL for thousand grain weight in European winter wheat (Triticum aestivum L.) by genome-wide association mapping. Front Plant Sci. 2015;6.

4. Ain Q, Rasheed A, Anwar A, Mahmood T, Imtiaz M, Mahmood T, et al. Genome-wide association for grain yield under rainfed conditions in historical wheat cultivars from Pakistan. Front Plant Sci. 2015;6.

5. Gao F, Liu J, Yang L, Wu X, Xiao Y, Xia X, et al. Genome-wide linkage mapping of QTL for physiological traits in a Chinese wheat population using the 90K SNP array. Euphytica. 2016;209:789–804.

6. Lin M, Zhang D, Liu S, Zhang G, Yu J, Fritz AK, et al. Genome-wide association analysis on pre-harvest sprouting resistance and grain color in U.S. winter wheat. BMC Genomics. 2016;17:794.

7. Gao L, Turner MK, Chao S, Kolmer J, Anderson JA. Genome Wide Association Study of Seedling and Adult Plant Leaf Rust Resistance in Elite Spring Wheat Breeding Lines. PLOS ONE. 2016;11:e0148671.

8. Dong Y, Liu J, Zhang Y, Geng H, Rasheed A, Xiao Y, et al. Genome-Wide Association of Stem Water Soluble Carbohydrates in Bread Wheat. PLOS ONE. 2016;11:e0164293.

9. Gao L, Zhao G, Huang D, Jia J. Candidate loci involved in domestication and improvement detected by a published 90K wheat SNP array. Sci Rep. 2017;7:44530.

10. Liu J, He Z, Rasheed A, Wen W, Yan J, Zhang P, et al. Genome-wide association mapping of black point reaction in common wheat (Triticum aestivum L.). BMC Plant Biol. 2017;17:220.

11. Wang S-X, Zhu Y-L, Zhang D-X, Shao H, Liu P, Hu J-B, et al. Genome-wide association study for grain yield and related traits in elite wheat varieties and advanced lines using SNP markers. PLOS ONE. 2017;12:e0188662.

12. Perez-Lara E, Semagn K, Tran VA, Ciechanowska I, Chen H, Iqbal M, et al. Population Structure and Genomewide Association Analysis of Resistance to Disease and Insensitivity to Ptr Toxins in Canadian Spring Wheat Using 90K SNP Array. Crop Sci. 2017;57:1522–39.

13. Wu J, Wang Q, Liu S, Huang S, Mu J, Zeng Q, et al. Saturation Mapping of a Major Effect QTL for Stripe Rust Resistance on Wheat Chromosome 2B in Cultivar Napo 63 Using SNP Genotyping Arrays. Front Plant Sci. 2017;8:653.

14. Shi W, Hao C, Zhang Y, Cheng J, Zhang Z, Liu J, et al. A Combined Association Mapping and Linkage Analysis of Kernel Number Per Spike in Common Wheat (Triticum aestivum L.). Front Plant Sci. 2017;8:1412.

15. Alomari DZ, Eggert K, von Wirén N, Pillen K, Röder MS. Genome-Wide Association Study of Calcium Accumulation in Grains of European Wheat Cultivars. Front Plant Sci. 2017;8.

16. Liu J, Feng B, Xu Z, Fan X, Jiang F, Jin X, et al. A genome-wide association study of wheat yield and quality-related traits in southwest China. Mol Breed. 2018;38:1.

17. Ma F, Xu Y, Ma Z, Li L, An D. Genome-wide association and validation of key loci for yield-related traits in wheat founder parent Xiaoyan 6. Mol Breed. 2018;38:91.

18. Alomari DZ, Eggert K, von Wirén N, Alqudah AM, Polley A, Plieske J, et al. Identifying Candidate Genes for Enhancing Grain Zn Concentration in Wheat. Front Plant Sci. 2018;9:1313.

19. Zuo J, Lin C-T, Cao H, Chen F, Liu Y, Liu J. Genome-wide association study and quantitative trait loci mapping of seed dormancy in common wheat (Triticum aestivum L.). Planta. 2019;250:187–98.

20. Sheoran S, Jaiswal S, Kumar D, Raghav N, Sharma R, Pawar S, et al. Uncovering Genomic Regions Associated With 36 Agro-Morphological Traits in Indian Spring Wheat Using GWAS. Front Plant Sci. 2019;10:527.
