# Supplementary figures and images for: Genome-wide association study of agronomic traits in winter wheat (Triticum aestivum L.) using a custom SNP marker set
Source: BMC Plant Biol. 2025 Oct 1;25:1279. doi: 10.1186/s12870-025-07322-y (PMC12487310; doi:10.1186/s12870-025-07322-y)

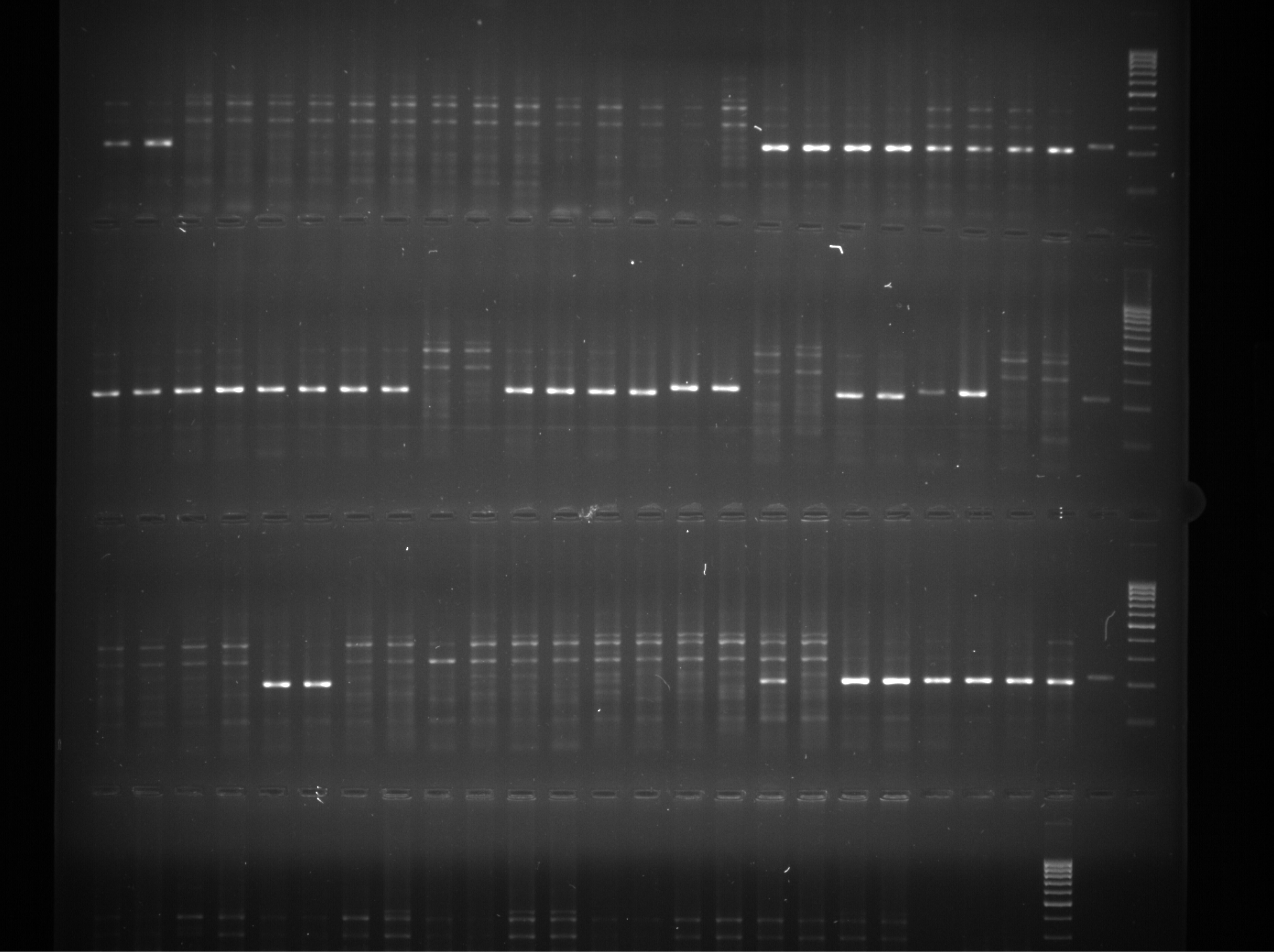

Supplement: Supplementary file 1 — Supplementary Material 1. Additional file 1.pdf supplementary figures and tables. Supplementary figure 1. Proportion of missing (no-call) values for each of the 16.5 K SNPs mapped on chromosomes pseudomolecules of RefSeq1.0 wheat genome assembly; Supplementary figure 2. Plots showing the distribution of SNPs among chromosomes and the gradual decrease their number on various stages of filtering; Supplementary figure 3. Geographical origin of winter wheat accessions on the scatterplot of principal components (PC1 – PC2); Supplementary figure 4. The geographical origin of wheat accessions shown on the neighbor-joining tree; Supplementary figure 5. Manhattan-plots and QQ-plots resulting from GWAS; Supplementary figure 6. Boxplots for significant marker-trait associations found in GWAS; Supplementary figure 7. KASP markers visualization; Supplementary table 1. A list of studies that were automatically screened for mentioning polymorphisms from wheat iSelect 90k array; Supplementary table 2. The KASP primers for detecting SNP alleles; Supplementary table 3. Comparison the KASP markers results and SNP-Seq. Additional file 2.xlsx Initial 60 K SNP set: positions and short descriptions of 60 thousand initial SNPs selected for SNP-seq marker panel design. Additional file 3.xlsx Oligonucleotide probes used for target DNA fragments enrichment. Additional file 4.xlsx Correspondence between sequencing samples and wheat accessions. Additional file 5.xlsx Phenotype BLUES used for GWAS. Additional file 6.xlsx Genotypes of 200 winter wheat accessions (16.497 SNP-seq markers). Additional file 7.xlsx 1RS.1BL translocation prediction and validation. Additional file 8.xlsx Diversity of specific sequencing-genotyped SNPs that are commonly used as breeding markers. Additional file 9.xlsx Significant marker-trait associations. [file 12870_2025_7322_MOESM1_ESM.zip › Gel image for Fig 3c.jpg]
